# PDMS Mixed Matrix Membrane with Confined Mass Transfer Structure: The Effect of COFs with Different Porous Structures and Chemical Properties in the Pervaperation Process

**DOI:** 10.3390/membranes15100316

**Published:** 2025-10-15

**Authors:** Yuan Zhai, Zimeng Zheng, Xinhao Cui, Kun Jiang, Ao Sheng, Heyun Wang

**Affiliations:** 1 School of Chemistry and Chemical Engineering, Shihezi University, Shihezi 832003, China; 20242007144@stu.shzu.edu.cn (Y.Z.); zhengzimeng2025@163.com (Z.Z.); cuixinhao@stu.shzu.edu.cn (X.C.); 20232107016@stu.shzu.edu.cn (K.J.); 20242007121@stu.shzu.edu.cn (A.S.); 2 Key Laboratory for Green Processing of Chemical Engineering of Xinjiang Bingtuan, Shihezi 832003, China; 3 Key Laboratory of Materials-Oriented Chemical Engineering of Xinjiang Uygur Autonomous Rigion, Shihezi 832003, China; 4 Engineering Research Center of Materials-Oriented Chemical Engineering of Xinjiang Bingtuan, Shihezi 832003, China

**Keywords:** pervaporation, confined mass transfer structure, mixed matrix membrane, covalent organic framework, simulation calculation

## Abstract

In this study, hydrophilic covalent organic framework (COF) nanosheets with triazine structures and hydrophobic COF nanosheets with fluorinated imine skeletons were designed to enhance the membrane separation process for ethanol pervaporation. The mass transfer of ethanol–water mixtures within the confined structures of COF nanosheets was investigated through experimental characterization and computational simulations, establishing a quantitative relationship between mass transfer performance and the pore size/chemical properties of COF nanosheets. These COF nanosheets were employed to optimize the confined architecture of mixed matrix membranes (MMMs), effectively regulating the critical parameters of MMMs and improving their separation performance. Through systematic investigation of formation mechanisms and modulation principles, we revealed the correlation between confined structural parameters and membrane separation efficiency. This work develops methodologies and foundational theories to overcome the permeability-selectivity trade-off effect, providing theoretical guidance for designing novel membrane materials with ethanol-permelective COF-based MMMs.

## 1. Introduction

Pervaporation membranes differ from ultrafiltration, nanofiltration, and other membrane separation processes that rely on macroporous matrices to screen materials based on pore size. Instead, pervaporation membranes feature apertures smaller than 2 nm, enabling confined mass transfer in spaces comparable to the free path of fluid molecules [1,2,3,4]. This confining effect significantly enhances the influence of fluid interactions with the previously negligible wall surface. The interactions among fluid molecules become comparable to those with the confining wall surface, which subsequently becomes a decisive factor in the transfer process. A stronger affinity between the walls and molecules accelerates molecular transport, a phenomenon known as the “confined mass transfer effect” [5,6]. The confined mass transfer process in pervaporation membranes exhibits unique mechanisms and intensification methods compared to macroscopic fluid transfer processes [7,8]. However, existing mass transfer models for confined separation membranes are often “black box” models that fail to quantitatively describe how membrane microstructure and pore interface chemistry influence flux and selectivity. This limited understanding of common mass transfer mechanisms and regulation methods for abnormal phenomena in confined structures hampers the design and fabrication of advanced membrane materials. To further explore the mass transfer principles of nano-confined fluids, various experimental methods have been employed, often using porous films, nanotube films, or graphene membranes with nanoporous channels for measurement [9,10,11]. However, due to material defects, these measurements often suffer from significant errors. Despite the rapid development of modern nano manufacturing, control, and measurement technology, there are still many difficulties in comprehensively understanding nano-confined fluid flow through experimental approaches alone [12,13]. As a result, numerical simulation methods are widely used in the study of nano-confined fluids. In recent years, experimental and molecular simulation studies have shown that pore size, surface properties, surface morphology, and pore modification all affect water transfer behavior in nanochannels [14,15,16,17].

Covalent Organic Frameworks (COFs) are crystalline porous materials composed of purely organic groups interconnected by covalent bonds, constructed from a variety of rigid organic structural units. With high thermochemical stability, large specific surface area and porosity, low skeletal density, controllable chemical and physical properties, permanently open pore structure, and diverse synthetic strategies, COFs have a wide range of potential applications in the fields of environment and energy [16,17,18,19,20]. COFs offer precise design capabilities for their structure, enabling different geometric configurations, including varied pore shapes and aperture sizes, unlike graphene [21,22,23]. Functional COF materials with unique physical and chemical properties can be obtained through the rational design and selection of building units. COFs also make an excellent nanoscale construction unit for confined mass transfer and separation channels. The maximum confined mass transfer effect can be achieved by tuning multiple interactions between COFs and permeating components, such as π-π electron donor-acceptor interaction, π-π stacking, hydrophobic interaction, van der Waals force, and hydrogen bond interaction through the adjustable and modifiable characteristics of COF structures [24,25].

The pervaporation process relies on the membrane as its core component. Different materials and structures for the membrane can greatly impact its permeability and selectivity, with significant differences between them. Recently, carbon-based separation membranes based on the confined mass transfer mechanism have been becoming a research hotspot [26,27]. Carbon-based separation membranes with a confined mass transfer effect can be constructed using different forms of carbon materials, including orderly arranged carbon nanotube membranes and layer-upon-layer graphene membranes. Due to their flexibility and high aspect ratio, constructing separation membranes with an orderly arrangement of carbon nanotubes is challenging construct separation membranes with an orderly arrangement of carbon nanotubes [28,29]. A layer-by-layer stack of graphene oxide sheets can be used to create confinement effects in nanoscale mass transfer channels [30,31]. However, the controllable preparation of graphene oxide, including precise regulation of the graphene oxide layer size, the neat construction of nano channels between layers, and the number of oxygen-containing groups and surface charge, remains difficult to achieve. Additionally, preparing large continuous quantities for industrial applications continues to pose significant challenges.

Mixed matrix membranes (MMMs) are a novel class of membrane materials with a polymer matrix as the continuous phase and a filler as the dispersed phase. The filler can optimize the confined structure of MMMs and effectively regulate membrane characteristic parameters (free volume, crystallinity, etc.), making it an effective way to enhance the separation performance of pervaporation membranes [21,32,33,34]. COFs with pore structures and strong adsorption abilities have potential as filling agents for modulating confined channel structure size and hole wall nature to create efficient mass transfer separation membranes [35,36,37]. The mechanism of confined structure formation and regularity of modulation, along with confined structure parameters and mixed matrix membrane separation performance, can help establish the relationship between the method and preliminary theory to overcome the “trade-off” effect [38].

Currently, the study of COF nanosheets as fillers for preparing mixed matrix membranes for organic separation is ongoing [39,40,41,42,43,44,45], and the relationship between the mass transfer mechanism of COF confined structures and their separation performance is rarely reported. In the development of new membrane materials, Computational simulation is first used for efficient and low-cost prediction of separation performance. This strategy reduces the blindness and waste of traditional trial-and-error experiments, thereby lowering costs and increasing efficiency. Xue et al. [46]. employed a chemical etching strategy using CuSO_4_ and HF to synthesize surface-porous 2D SPMXene nanosheets from 2D MXene. Molecular dynamics simulations of three membranes indicate that etched surface pores on 2D SPMXene nanosheets enlarge free volume and create extra channels, raising water permeability.

In this paper, COF materials with different properties were used to carry out vapor adsorption experiments of water and ethanol, evaluating the difficulty of molecular entry into COF channels, to construct the skeleton structure of these four COFs through simulation calculations, to simulate the mass transfer process of water and ethanol in these four COF channels and the changes in simulation calculation forces, and to combine the experimental data with the simulation calculation data. The analysis aimed to determine the influence of the differences among the four COF materials on the pervaporation separation performance, to explore the mass transfer mechanisms of ethanol and water in these four COFs, to further explore the mass transfer mechanisms of membrane separation in mixed matrix membranes, and to construct the best mass transfer model.

## 2. Methodology

### 2.1. Materials

All chemicals were purchased without further modification. Shenzhen hongyejie Technology Co., Ltd. (Shenzhen, China): Polydimethylsiloxane (PDMS 5000 mPa.s); Shanghai Aladdin Industrial Corporation (Shanghai, China): 1,4-Phthalaldehyde (98%), Triethylamine (97%), Heptane (98%), Dibutyltin dilaurate (DBTDL, 98%), Ethanol absolute (99%), 1-Butanol (99%), 1,2-dichlorobenzene (98%), Melamine (98%), Cyanuric chloride (98%), Tetraethyl orthosilicate(TEOS, 98%); Jilin zhongxueshen Technology Co., Ltd. (Jilin, China): tetrafluoro-p-benzaldehyde (97%), 1,3,5-triaminobenzene trihydrochloride (98%),1,3,5-tris (4-aminophenyl) benzene (98%); Zhongke Ruiyang Co., Ltd. (Beijing, China): Polyethersulfone (PES, 30000 retained molecular weight); Tianjin Fuyu Co., Ltd. (Tianjin, China): methanol (99.5%), Tetrahydrofuran (99%), DMSO (99%); School controlled hazardous chemicals: sodium, Self-made in laboratory: Deionized water.

### 2.2. Experimental Part

**Figure 1:**
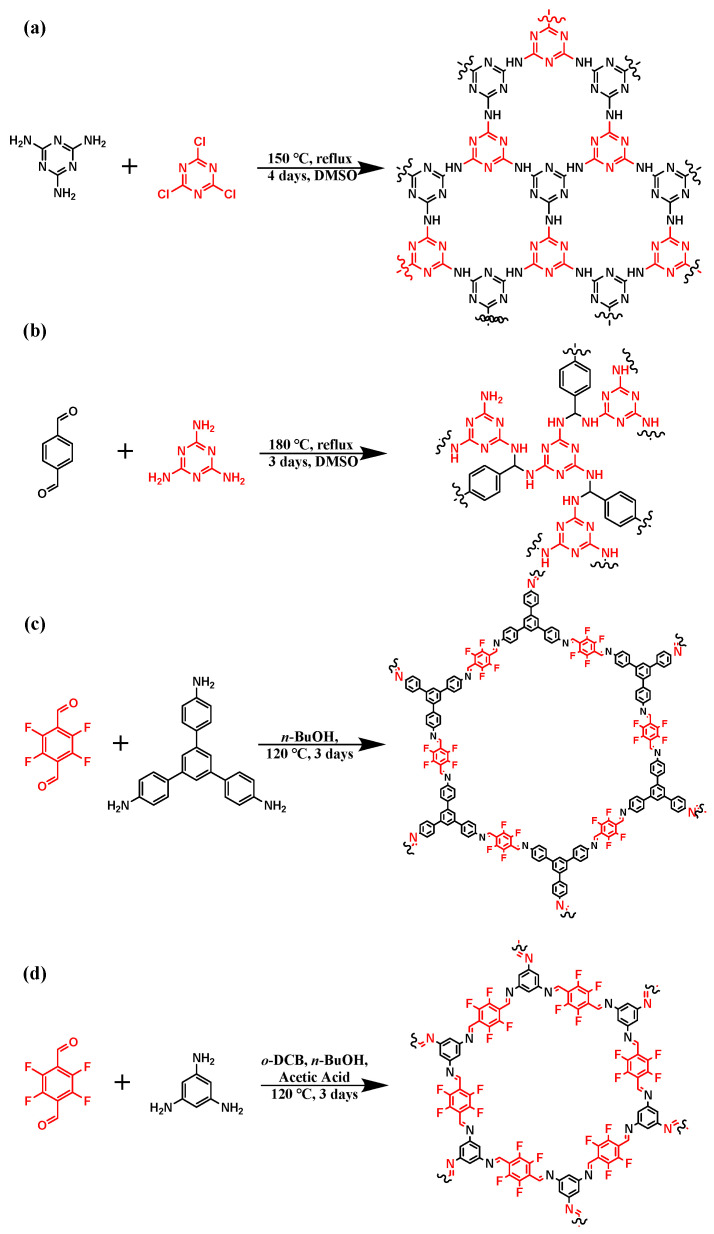
The mechanism of the (**a**) NENP-1 (**b**) SNW-1 (**c**) SCF-FCOF-2 (**d**) SCF-FCOF-2x reaction.

We synthesized four distinct types of COFs, NENP-1, SNW-1, SCF-FCOF-2 and SCF-FCOF-2x. The reaction mechanism is illustrated in Figure 1. Detailed experimental procedures are provided in the Supporting Information.

### 2.3. Vapor Adsorption Experiment

The adsorption properties of water and ethanol on four different COFs were investigated using a Steam-1010 vapor sorption analyzer. Approximately 0.1 g of each COF sample was weighed and placed into the sample tube of the analyzer. The tube was subsequently degassed at 120 °C for 6 h, after which the mass of the degassed sample was measured. Following degassing, the sample tube was connected to the vapor sorption analyzer, the sample weight was calibrated, and the steam adsorption module was activated. Prior to initiating the test, the analyzer was preheated, and the ambient adsorption temperature was set to 35 °C. The cabin temperature in the steam generator was adjusted to 70 °C for water vapor and 65 °C for ethanol vapor. Once the temperatures were stabilized, the steam adsorption test was commenced.

### 2.4. Establishment of COFs Crystal Model

**Figure 2:**
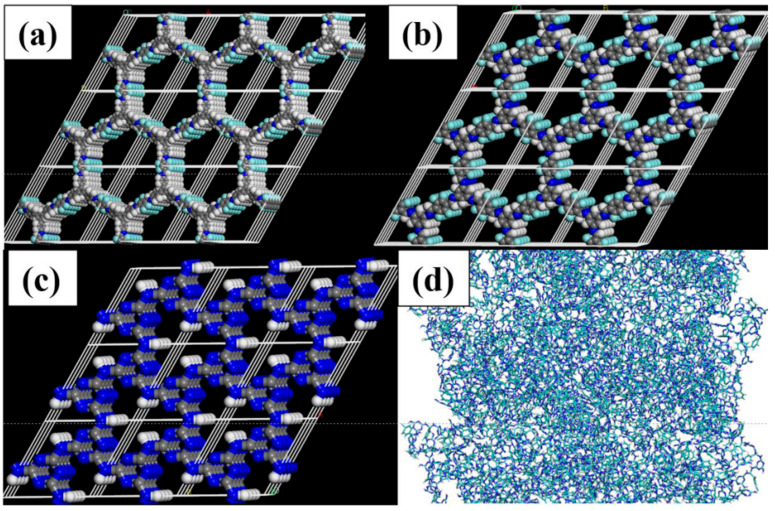
Four models of COFs: (**a**) SCF-FCOF-2, (**b**) SCF-FCOF-2x, (**c**) NENP-1, (**d**) SNW-1.

The crystal models of three COFs, namely NENP-1, SCF-FCOF-2, and SCF-FCOF-2x, were constructed using Materials Studio 8.0 software. Given the topological nature of COFs, which typically comprise two or more repeating units, simplified periodic structures were generated as follows. Firstly, the repeating unit of each COF was drawn and geometrically optimized using the Dmol^3^ module. Then, a simple hexagonal cell was constructed and enlarged. The chemical structure of the COFs was mimicked, and the repeating units in each cell were connected via covalent bonds. Subsequently, the overall force field of the structure was optimized using the Forcite module to find the best symmetry and apply it to the cell. The overall force field was further refined using the Forcite module to obtain the initial structure of the three COFs. The crystal structure was parameterized using PRODRG and processed with the GROMOS force field. The atomic charges were calculated using Gaussian quantification of the electronic density, and the crystal model was further optimized to finalize complete models for the three COFs. As the chemical structure of SNW-1 is difficult to construct in the same plane, building a 3D model using Materials Studio software made it challenging. Therefore, GROMACS 4.05 software was used to directly simulate the cross-linking aggregation process of SNW-1 to obtain its chemical model. By setting the ratio of melamine to benzene-1,4-dialdehyde monomers to 2:3, the system’s charge balance could be better maintained, and a model with structure and properties similar to experimentally synthesized SNW-1 could be obtained. 12 benzene-1,4-dialdehyde molecules and 8 melamine molecules were randomly inserted into a simulation box with a dimension of 4 nm, and Dynamic cross-linking was performed for 4 ns under vacuum conditions in the NVT ensemble at 460 K to obtain a complete SNW-1 model. This protocol ensured charge balance and yielded a model consistent with experimentally synthesized SNW-1 (Figure 2).

### 2.5. Mass Transfer Process Simulation

All COF crystal structures were expanded into 3D architectures by fabricating continuous membranes; dimensional parameters are shown in Table 1. Atomic coordinates were fixed, and force field parameters were optimized using the GROMOS force field to simulate water/ethanol transport. Each membrane was positioned at the base of a cubic simulation cell (edge length: 5 nm), with water and ethanol molecules placed above the membrane surface. Periodic boundary conditions were applied in all three dimensions to approximate bulk-phase behavior. Simulations utilized the NVT ensemble (constant particle number, volume, and temperature), with the temperature maintained at 300 K via a Berendsen thermostat. Production runs exceeded 1 ns using a 2 fs time step to ensure equilibration of molecular trajectories.

**Figure 3:**
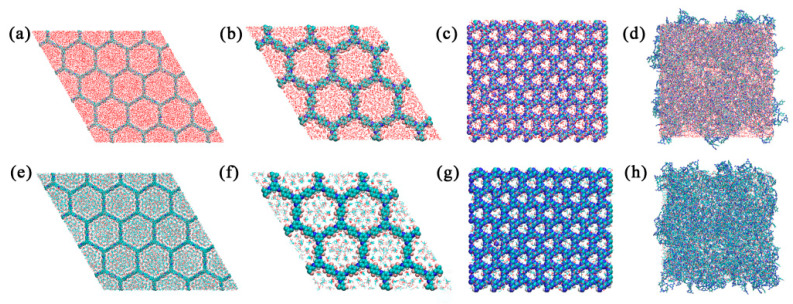
The water molecule mass transfer model of (**a**) SCF-FCOF-2, (**b**) SCF-FCOF-2x, (**c**) NENP-1, (**d**) SNW-1; The ethanol molecule mass transfer model of (**e**) SCF-FCOF-2, (**f**) SCF-FCOF-2x, (**g**) NENP-1, (**h**) SNW-1.

Nonbonded interactions between permeate molecules and COF membranes were quantified, enabling the calculation of intra- and inter-membrane diffusion coefficients. Visualization of mass transfer pathways (Figure 3) and dynamic interactions was performed using VMD, revealing preferential molecular trajectories through COF pores compared to PDMS free volumes.

## 3. Results and Discussion

### 3.1. Vapor Adsorption Properties of Hydrophilic COFs and Hydrophobic COFs

After ethanol and water molecules enter the mixed matrix membrane through dissolution, their diffusion occurs via two pathways: one through the free volume between PDMS polymer chains, and the other through the COF pores. To further investigate the ease of diffusion for ethanol and water molecules into the COF pores indicating the solubility selectivity of COF pores toward ethanol-adsorption experiments were first conducted for water vapor and ethanol vapor on amphiphilic COF materials. As shown in Figure 3a and Table 2, at P/P_0_ < 0.2, both NENP-1 and SNW-1 exhibited ethanol-preferential adsorption. NENP-1 showed moderate ethanol selectivity, with ethanol uptake 1.4× higher than water at P/P_0_ = 0.1, attributed to its balanced triazine- and amino-functionalized pores. With a BET surface area of 302 m^2^/g and pore size of 1.4 nm (Figure S2), its hydrophilic groups create comparable affinity for both molecules, limiting preferential adsorption. However, ethanol uptake surpasses water at higher pressures likely, due to steric hindrance from the triazine rings restricting water multilayer formation. In contrast, SNW-1 demonstrated pronounced ethanol selectivity, achieving a 2.6× higher ethanol adsorption under identical conditions, driven by its ultra-microporous structure (0.7 nm) and high surface area (674 m^2^/g, Figure S6). The benzene-rich framework enhances π-π interactions with ethanol, with the increase in pressure, the adsorption capacity of ethanol was always higher than that of water. Compared with water molecules, ethanol molecules were more easily preferentially absorbed into the inner channel of SNW-1. Despite its hydrophilicity, the narrow pore size limits water capillary condensation, favoring ethanol retention. The results showed that ethanol had stronger specific adsorption in the condition of hydrophilic.

**Figure 4:**
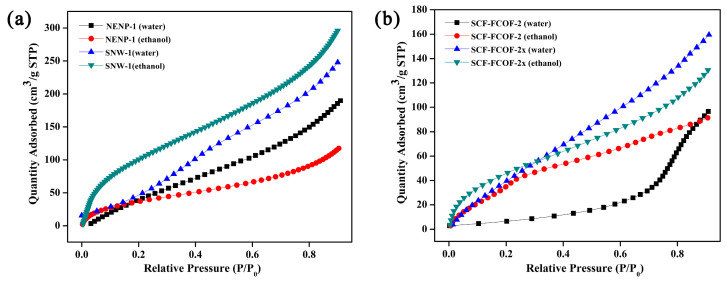
Vapor adsorption performance of (**a**) hydrophilic COFs, (**b**) Hydrophobic COFs.

Subsequent vapor adsorption experiments on hydrophobic COFs (Figure 4b, Table 2) revealed distinct pressure-dependent behaviors. SCF-FCOF-2′s adsorption behavior is governed by its fluorinated framework and pore architecture. With a BET surface area of 224 m^2^/g and a pore size of 3.1 nm (Figure S10), its large channels allow ethanol and water molecules to access the pores, while the fluorine groups significantly reduce surface energy, suppressing initial adsorption at low pressures. Notably, SCF-FCOF-2 demonstrated significant ethanol selectivity at P/P_0_ = 0.1, with ethanol uptake 4.3× higher than water. However, residual benzene motifs in the structure facilitate selective ethanol adsorption through hydrophobic π-π interactions as pressure increases. At P/P_0_ = 0.9, ethanol uptake reaches 91.4 cm^3^/g via multilayer adsorption, whereas water adsorption surges to 96.5 cm^3^/g due to capillary condensation in the larger pores. This dichotomy highlights the interplay between fluorine-induced hydrophobicity and pore size-driven capillary effects.

In contrast, SCF-FCOF-2x exhibits altered adsorption dynamics due to structural modifications. The introduction of amino/imino groups partially offsets the hydrophobicity of the fluorinated framework (contact angle reduced by 22° compared to SCF-FCOF-2, Figures S8 and S16), while the narrowed pore size (1.4 nm, BET 218 m^2^/g, Figure S14) imposes steric constraints. At low pressure (P/P_0_ = 0.1), ethanol selectivity diminishes, as polar amino groups compete with π-π interactions. However, at P/P_0_ > 0.7, water co-adsorption increases significantly, likely due to hydrogen bonding with amino functionalities. Ethanol diffusion is further restricted by the smaller pores, limiting its multilayer accumulation. These results underscore how pore chemistry and size synergistically modulate adsorption equilibrium in hydrophobic COFs. Collectively, these adsorption studies demonstrate that pore affinity and structural composition critically govern the diffusion selectivity of ethanol over water in COFs. This work establishes that tailoring pore chemistry and surface energy control are pivotal for engineering COF-based membranes with targeted molecular sieving capabilities.

### 3.2. Simulation of Mass Transfer of Water and Ethanol Molecules in Four Different COFs

The mass transfer of ethanol and water in COFs involves dissolution (adsorption) and diffusion processes. However, the adsorption performance of the four COF fillers for ethanol/water vapor alone cannot fully elucidate the pervaporation separation mechanism. To address this, molecular dynamics simulations using the GROMACS package were employed to analyze diffusion dynamics within these COFs. As shown in Table 3, electrostatic interactions-primarily hydrogen bonding between water/ethanol molecules and COF pore structures were quantitatively assessed. NENP-1 exhibits significantly stronger electrostatic interactions with water than ethanol. This preferential adsorption impedes water diffusion, thereby enhancing the separation efficiency of the NENP-1/PDMS membrane. In contrast, SNW-1 shows a smaller differential in electrostatic forces, with water interactions measured at 992.6% of ethanol interactions and ethanol interactions at 345.2% of water interactions. This reduced disparity, combined with stronger absolute forces on both components, increases diffusion resistance and slows mass transfer rates, explaining its inferior selectivity compared to NENP-1. For SCF-FCOF-2 and SCF-FCOF-2x exhibit less pronounced electrostatic disparities between water and ethanol, suggesting their influence is predominantly on dissolution thermodynamics rather than diffusion kinetics in mixed matrix membranes. These computational insights align with experimental observations, demonstrating the critical role of electrostatic interactions in governing separation performance.

To elucidate the diffusion dynamics of ethanol and water within NENP-1, SNW-1, SCF-FCOF-2, and SCF-FCOF-2x, molecular diffusion coefficients (D) within the COF pores were quantified (Table 4). For hydrophilic COFs, NENP-1 exhibited significantly reduced water diffusion coefficients compared to ethanol, highlighting its ability to selectively hinder water diffusion while minimally impeding ethanol transport. This pronounced disparity in diffusivity is critical to the superior separation performance of the NENP-1/PDMS membrane. The enhanced selectivity arises from NENP-1’s triazine-rich pore architecture and amino functional groups, which strengthen water-specific hydrogen bonding interactions, thereby amplifying diffusion selectivity. In contrast, SNW-1 showed lower ethanol diffusivity and weaker discrimination between components, aligning with its reduced separation efficiency. Both COFs displayed less restrictive diffusion behavior, with water diffusing faster than ethanol due to its smaller molecular size, supporting the hypothesis that hydrophobic COFs primarily govern dissolution thermodynamics rather than diffusion kinetics. Notably, SCF-FCOF-2x exhibited further reduced ethanol diffusivity, attributed to its narrower pore dimensions and additional amino groups, which introduce steric hindrance and electrostatic barriers to ethanol transport.

### 3.3. Optimization of Alcohol Mass Transfer Model

Based on the distinct adsorption behaviors of water and ethanol vapors across four COFs and mechanistic insights into their mass transfer properties, a systematic comparative study of both pristine COFs and their corresponding MMMs was conducted by integrating existing experimental datasets in Figure S32. As summarized in Table 5, NENP-1 demonstrates a synergistic balance between solubility selectivity and enhanced diffusivity discrimination, collectively contributing to the superior separation performance of the NENP-1/PDMS membrane. SCF-FCOF-2 exhibits a complementary mechanism dominated by pronounced solubility selectivity coupled with moderate diffusion selectivity, yielding MMMs with high permeance and stable separation efficiency. In contrast, SNW-1, despite its exceptional solubility selectivity, shows compromised performance due to inverted diffusion selectivity and elevated diffusion resistance, which reduce both flux and separation factor. The SCF-FCOF-2x variant displays further deterioration, with diminished solubility selectivity and unfavorable diffusivity characteristics, resulting in suboptimal MMM performance. Experimental trends align with simulations, confirming theoretical models’ ability to predict physicochemical behavior.

## 4. Conclusions

Membrane mass transfer processes are intrinsically complex, particularly in mixed matrix membranes (MMMs) incorporating fillers. A comprehensive understanding of MMM mass transfer mechanisms necessitates examining not only solubility-diffusion processes within the polymer matrix but also those occurring in the fillers. This study combines vapor adsorption experiments with computational simulations to model water/ethanol solubility-diffusion behavior in four COF materials. Through integrated analysis of experimental and simulation data, we elucidate how COFs govern mass transfer in pervaporation processes, revealing distinct solubility-diffusion pathways among the COFs. These analyses reveal material-specific transport mechanisms: hydrophilic NENP-1 facilitates ethanol/water separation through its abundant triazine rings and amino groups, which generate 992.6% stronger electrostatic interactions with water molecules than with ethanol molecules. This preferential binding effectively hinders water transport across the membrane. In contrast, the larger pore size of NENP-1 (1.4 nm) compared to that of SNW-1 (0.7 nm) resulted in a higher ethanol diffusion rate. Conversely, hydrophobic COFs predominantly influence separation by modulating solubility selectivity at membrane surfaces. SCF-FCOF-2 and SCF-FCOF-2x possess similar structures and thus analogous dissolution-diffusion characteristics, their separation performance is decided by pore size. This distinction is further exemplified by SCF-FCOF-2x (1.4 nm): its smaller pore dimensions compared to SCF-FCOF-2 (3.1 nm), combined with additional amino groups within pore channels, collectively impede ethanol diffusion. Building on these mechanistic insights, we propose a composite membrane architecture: A hydrophobic COF/PDMS MMM as the feed-facing layer controls solubility selectivity, while a hydrophilic COF/PDMS MMM filtration layer governs diffusion selectivity. This bilayer design theoretically enables superior separation performance compared to one-layer MMMs.

## Figures and Tables

**Table 1:** The size of the four COFs models.

Classification	Length (nm)	Width (nm)	Height (nm)	Volume (nm^3^)
SCF-FCOF-2	15.12	13.09	13.09	2590.93
SCF-FCOF-2x	6.83	5.91	8.26	333.57
NENP-1	6.9	5.97	6.77	278.76
SNW-1	7.79	7.79	3.46	209.88

**Table 2:** Adsorption performance of COFs for water and ethanol.

P/P_0_	NENP-1		SNW-1		SCF-FCOF-2		SCF-FCOF-2x	
	Water (cm^3^g^−1^)	Ethanol (cm^3^g^−1^)	Water (cm^3^g^−1^)	Ethanol (cm^3^g^−1^)	Water (cm^3^g^−1^)	Ethanol (cm^3^g^−1^)	Water (cm^3^g^−1^)	Ethanol (cm^3^g^−1^)
0.1	20.3	27.5	28.6	75.4	4.7	20.0	23.6	32.7
0.2	37.8	36.9	49.4	100.6	6.6	34.8	39.6	46.2
0.3	56.9	44.3	70.9	122.3	8.6	46.6	56.3	55.7
0.5	89.8	59.1	132.9	163.5	15.4	61.3	87.3	74.9
0.7	123.5	76.7	173.8	209.7	31.4	76.2	114.7	94.5
0.9	189.6	117.3	247.9	296.0	96.5	91.4	159.5	130.4

**Table 3:** The interaction between water and ethanol on different COFs.

Model	Electrostatic Force (nm^−3^)		Electrostatic Force Ratio (Water/Ethanol)
	Water	Ethanol	
NENP-1	−120.1	−12.1	992.6%
SNW-1	−1054.6	−305.5	345.2%
SCF-FCOF-2	−48.7	−33.2	146.7%
SCF-FCOF-2x	−103.0	−57.7	178.5%

**Table 4:** Diffusion coefficients of ethanol and water in different media.

Media	Diffusion Coefficient (1 × 10^−5^ cm^2^s^−1^)	
	Water	Ethanol
NENP-1	0.004026 (±0.00295)	3.4610 (±0.1017)
SNW-1	0.2158 (±0.0404)	0.4879 (±0.2050)
SCF-FCOF-2	3.1769 (±0.0258)	0.8553 (±0.2917)
SCF-FCOF-2x	2.7561 (±0.7002)	0.5128 (±0.3604)

**Table 5:** Comparison of COFs simulation data and experimental data.

Media	Dissolution Selectivity	Diffusion Selectivity	Total Flux of MMMs (g∙m^−2^h^−1^)	Separation Factor of MMMs
NENP-1	Excellent	Excellent	596	17.1
SNW-1	Medium	Medium	422	14.7
SCF-FCOF-2	Excellent	Excellent	605	16.8
SCF-FCOF-2x	Bad	Bad	223	8.2

## Data Availability

The original contributions presented in this study are included in the article and supplementary material. Further inquiries can be directed to the corresponding author.

## Supplementary Materials

The following supporting information can be downloaded at: https://www.mdpi.com/article/10.3390/membranes15100316/s1, Section S1: Experimental procedures; Section S2: Results and Discussion; Section S3: Characterization of COFs/PDMS MMMs; Section S4: Pervaporation Performance of COFs/PDMS MMMs.
