# Supplementary material for: PDMS Mixed Matrix Membrane with Confined Mass Transfer Structure: The Effect of COFs with Different Porous Structures and Chemical Properties in the Pervaperation Process
Source: Membranes (Basel). 2025 Oct 15;15(10):316. doi: 10.3390/membranes15100316 (PMC12566124; doi:10.3390/membranes15100316)
Supplement: Supplementary file 1 [file membranes-15-00316-s001.zip › membranes-3883910-supplementary.pdf]

Supporting Information

PDMS mixed matrix membrane with confined mass transfer structure: the effect of COFs with different porous structure and chemical property in pervaperation process

Yuan Zhai [1], Zimeng Zheng [1], Xinhao Cui [1], Kun Jiang [1], Ao Sheng [1] and Heyun Wang [1,2,3,4,*]

1 School of Chemistry and Chemical Engineering, Shihezi University, Shihezi 832003, China; 20242007144@stu.shzu.edu.cn (Y.Z.); zhengzimeng2025@163.com (Z.Z.); cuixinhao@stu.shzu.edu.cn (X.C.); 20232107016@stu.shzu.edu.cn (K.J.); 20242007121@stu.shzu.edu.cn (A.S.)

2 Key Laboratory for Green Processing of Chemical Engineering of Xinjiang Bingtuan, Shihezi 832003, China

3 Key Laboratory of Materials-Oriented Chemical Engineering of Xinjiang Uygur Autonomous Shihezi 832003, China

4 Engineering Research Center of Materials-Oriented Chemical Engineering of Xinjiang Bingtuan, Shihezi 832003, China

* Correspondence: whyun@shzu.edu.cn; Tel.: +8618609932991; Fax: +86-0993-2057272

S1. Experimental procedures

Materials

All chemicals were purchased without further modification. Shenzhen hongyejie Technology Co., Ltd: Polydimethylsiloxane (PDMS 5000 mPa.s); Shanghai Aladdin Industrial Corporation: 1,4-Phthalaldehyde (98%), Triethylamine(97%), Heptane (98%), Dibutyltin dilaurate (DBTDL, 98%), Ethanol absolute (99%), 1-Butanol (99%), 1,2-dichlorobenzene (98%), Melamine (98%), Cyanuric chloride (98%), Tetraethyl orthosilicate(TEOS, 98%); Jilin zhongxueshen Technology Co., Ltd: tetrafluoro-p-benzaldehyde (97%),1,3,5-triaminobenzene trihydrochloride (98%), 1,3,5-tris (4-aminophenyl) benzene (98%); Zhongke Ruiyang Co., Ltd: Polyethersulfone (PES, 30000 retained molecular weight); Tianjin Fuyu Co., Ltd: methanol (99.5%), Tetrahydrofuran (99%), DMSO (99%); School controlled hazardous chemicals: sodium, Self-made in laboratory: Deionized water.

Synthesis of NENP-1 filler

First, melamine (5 mmol, 0.6306 g) was dissolved in 25 mL DMSO, and cyanuric acid (5 mmol, 0.9221 g) was dissolved in another 25 mL of DMSO. The two solutions were sonicated for 30 minutes to ensure complete dissolution. After sonication, the solutions were combined in a 100 mL round-bottom flask and heated under stirring and reflux at 150 ℃ for 96 hours using an oil bath. After the reaction, the stirrer was switched off, and the mixture was left under reflux at 150 ℃ for an additional 12 hours, then allowed to cool slowly to room temperature in the oil bath.The resulting white solid was filtered through an organic phase filter membrane (0.22 um pore size) and washed sequentially with DMSO, deionized

water, and methanol to remove unreacted monomers. The product was then dispersed in 500 mL anhydrous methanol and stirred at room temperature for three days, with daily replacement of methanol to remove any residual DMSO and water. Afterward, the product was filtered through a 0.22 μm organic phase filter membrane and dried using supercritical carbon dioxide drying (2 hours per cycle, three cycles) in a wet state, resulting in a white powder with a yield of 69%.

Synthesis of SNW-1 filler

Melamine (313 mg, 2.485 mmol) and benzaldehyde (500 mg, 3.728 mmol) were dissolved in 15.5 mL DMSO in a Schlenk flask. The mixture was degassed by bubbling argon gas for 5 minutes, followed by three freeze-pump-thaw cycles. After degassing, the upper end of the condenser was sealed with a balloon, and the valve on the Schlenk flask was closed to create an inert reaction system.The reaction mixture was heated and stirred under reflux at 180 °C in an oil bath for 72 hours. After completion, the mixture was allowed to cool to room temperature, then filtered through a 0.22 μm filter membrane and washed with DMF and THF. The product was further purified by Soxhlet extraction using methanol, followed by THF, and then methanol again. Finally, the product was dried using supercritical carbon dioxide drying (2 hours per cycle, three cycles), resulting in a light yellow powder with a yield of 73%.

Synthesis of SCF-FCOF-2 filler

82.4 mg (0.400 mmol) of 4,4'-difluorobenzophenone and 93.6 mg (0.267 mmol) of 1,3,5-tris(4-aminophenyl) benzene were added to a 2 mL ampoule. 1 mL of anhydrous

n-butanol was then injected into the ampoule. The mixture was sonicated for 30 minutes to ensure complete dispersion of the components. After sonication, the mixture was subjected to three freeze-pump-thaw cycles to remove oxygen, and the ampoule was flame-sealed under vacuum after freezing.The sealed ampoule was heated in an oven at 120 ℃ for 72 hours. After the reaction, the ampoule was allowed to cool to room temperature, broken open, and the contents were extracted. The resulting mixture was filtered through a 0.22 μm PTFE membrane and washed several times with anhydrous tetrahydrofuran (THF) to remove any unreacted monomers and solvents.Next, the product underwent Soxhlet extraction with anhydrous THF for 12 hours, followed by soaking in 500 mL of anhydrous THF to replace the solvent, with daily solvent replacements to remove residual small molecules and water from the pores. Finally, the product was dried using supercritical carbon dioxide drying for 2 hours per cycle (three cycles), yielding an orange powder (yield 72%).

Synthesis of SCF-FCOF-2x filler

82.4 mg (0.400 mmol) of 4,4'-difluorobenzophenone and 62.08 mg (0.267 mmol) of 1,3,5-triaminobenzene trihydrochloride were added to a 2 mL ampoule. 0.9 mL of o-dichlorobenzene and 0.1 mL of n-butanol were then injected into the ampoule. The mixture was sonicated for 30 minutes to ensure uniform dispersion of the components. Subsequently, 0.1 mL of 6 M acetic acid and triethylamine were added, and the solution was subjected to three freeze-pump-thaw cycles to remove oxygen. After freezing, the ampoule was flame-sealed under vacuum. The sealed ampoule was heated in an oven at 120 °C for 72 hours. After the reaction, the ampoule was cooled to room temperature, opened, and the

mixture was extracted. The resulting mixture was filtered through a 0.22 μm PTFE membrane and washed several times with deionized water, anhydrous methanol, and anhydrous THF in sequence to remove any unreacted monomers and solvents.The product was further purified by Soxhlet extraction using anhydrous THF overnight. After Soxhlet extraction, the product was soaked in 500 mL of anhydrous THF for solvent replacement, with daily replacements of the anhydrous THF to remove small molecules and water from the pores. Finally, the product was dried three times using supercritical carbon dioxide drying for 2 hours per cycle, yielding a brown powder (yield 66%).

Synthesis of COFs/PDMS MMMs

First, mix PDMS and n-octane in a 1:1 mass ratio at room temperature and stir for 8 hours to obtain a uniform PDMS solution. Then, add a certain amount of COFs to n-octane, seal the container, and subject the mixture to ultrasonic dispersion for 2 hours followed by stirring for 4 hours. Repeat this cycle three times to achieve a well-dispersed COFs suspension. Combine the two mixed solutions in the specified proportions and stir for 0.5 hours, then add a certain amount of tetraethyl orthosilicate (TEOS) and continue stirring for another 0.5 hours. Finally, introduce a certain amount of dibutyltin dilaurate (DBTDL) as a catalyst to obtain a casting solution with component ratios of n-octane:PDMS:TEOS:DBTDL=500:100:10:1, and stir in an open system until the solution becomes viscous. Use a spin coater to evenly spread the casting solution onto a dry PES support layer. Place the mixed matrix membrane on a dust-free and dry horizontal film-forming platform and dry it for 6 hours at room temperature. Afterwards, heat the

membrane in a vacuum drying oven at 80 ℃ for another 6 hours to further complete the crosslinking process and evaporate the remaining solvent. Through testing and screening, the mass ratios of COFs to PDMS in the prepared mixed matrix membrane are determined to be 1 wt.%, 3 wt.%, 5 wt.%, and 7 wt.%.

S2. Results and Discussion

Characterization of NENP-1 filler

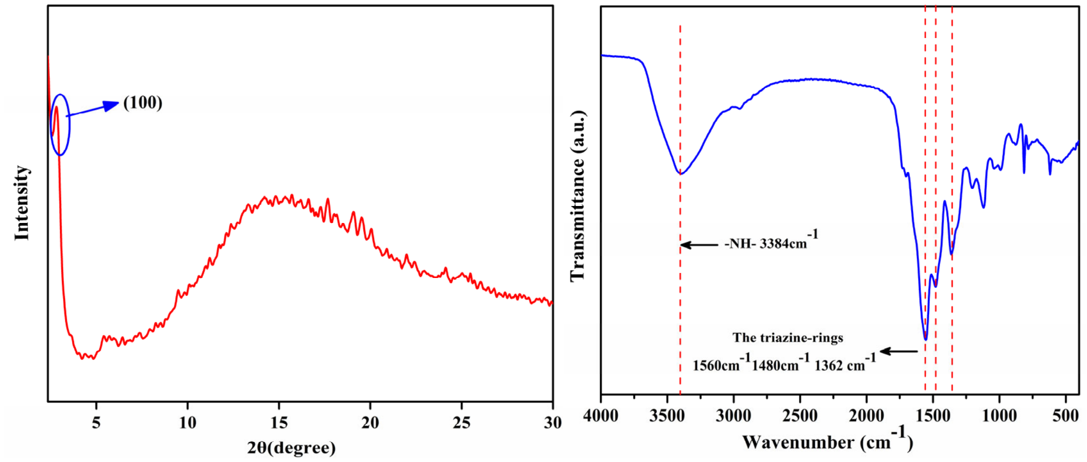

Fig.S1 XRD spectra and of FTIR spectra of NENP-1 nanoparticles

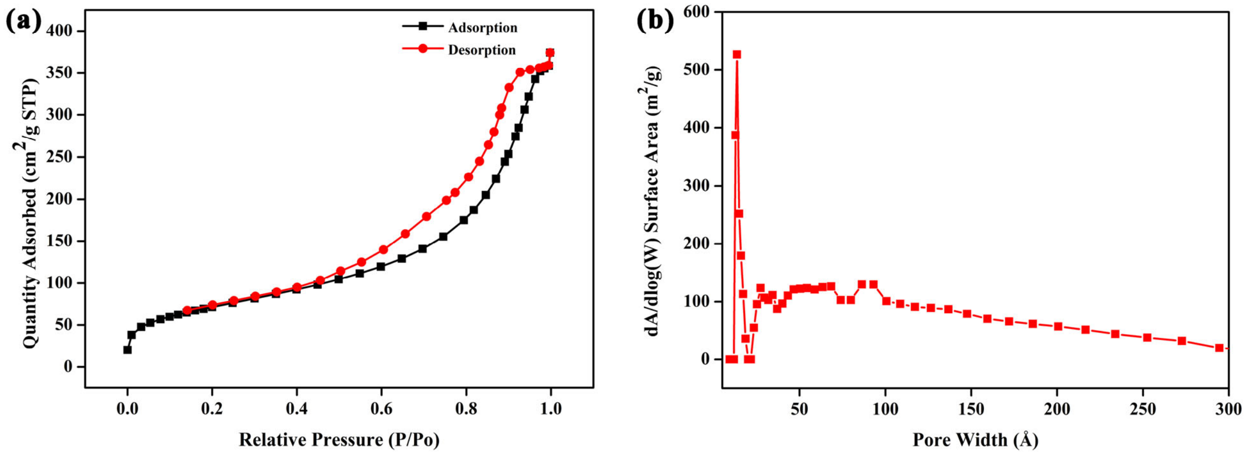

Fig.S2 (a) N_2 adsorption-desorption isotherms, (b) Pore size distribution histogram of NENP-1

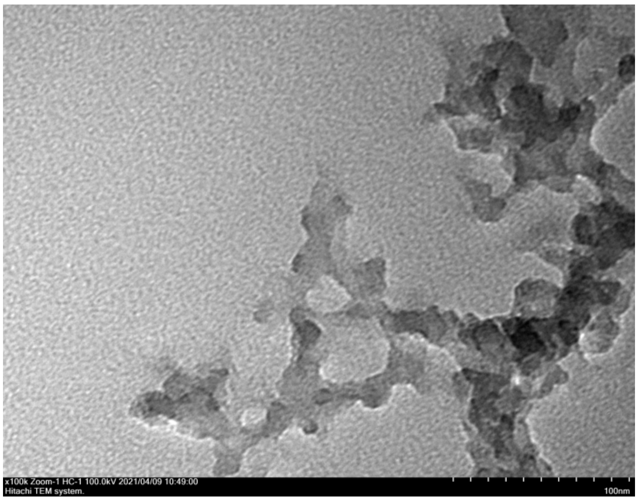

Fig. S3 TEM images NENP-1 nanoparticles

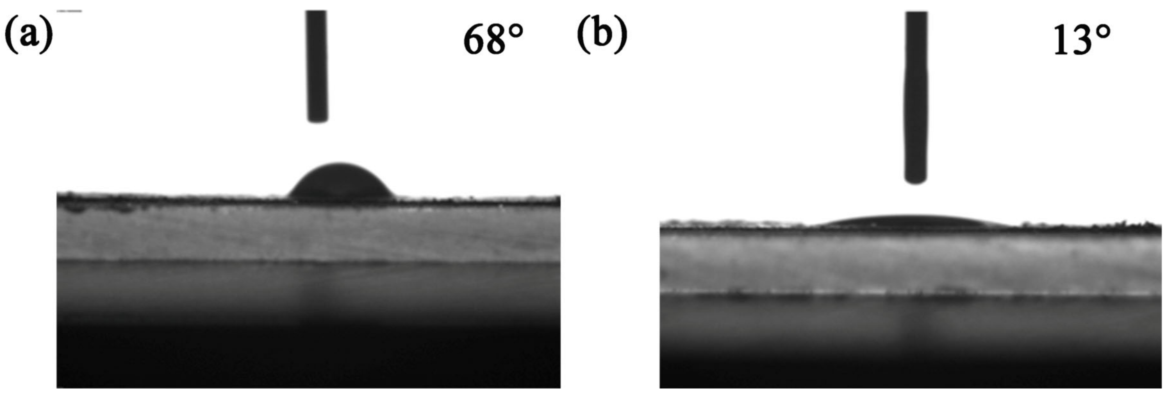

Fig. S4 (a) Water contact angle, (b) Ethanol contact angle of NENP-1 nanoparticles

Firstly, NENP-1 was characterized by XRD, and the characterization results are shown in **Fig.S1**. At 2.68°, there was a distinct (100) crystal surface characteristic peak, which provided preliminary evidence for the synthesis of NENP-1. Subsequently, the chemical structure of NENP-1 was investigated by FTIR. The characteristic peak at 3384 cm^{-1} represented the stretching vibration peak of the -NH group, while the peaks at 1560 cm^{-1}, 1480 cm^{-1}, and 1362 cm^{-1} represented the stretching vibration peaks of the triazine ring. BET characterization of NENP-1 was carried out as shown in **Fig.S2**, showing that the specific surface area of NENP-1 measured 302 m^2/g, with a pore size of about 1.4 nm. **Fig.S3** shows that NENP-1 has a lamellar structure, and the contact angle test in **Fig.S4** shows that NENP-1 has an affinity for both ethanol and water. This is due to the presence of a large number of triazine rings and amino groups.

Characterization of SNW-1 filler

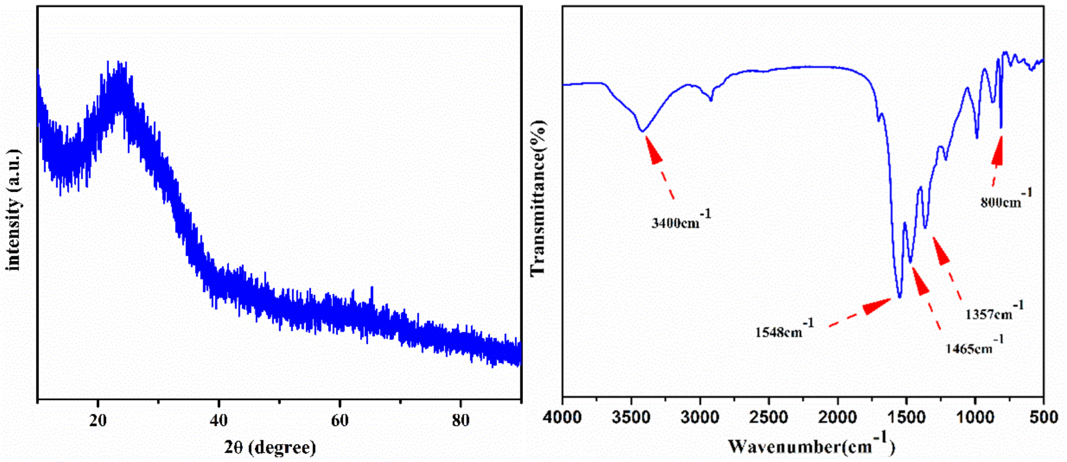

Fig.S5 XRD spectra and FTIR spectra of SNW-1 nanoparticles

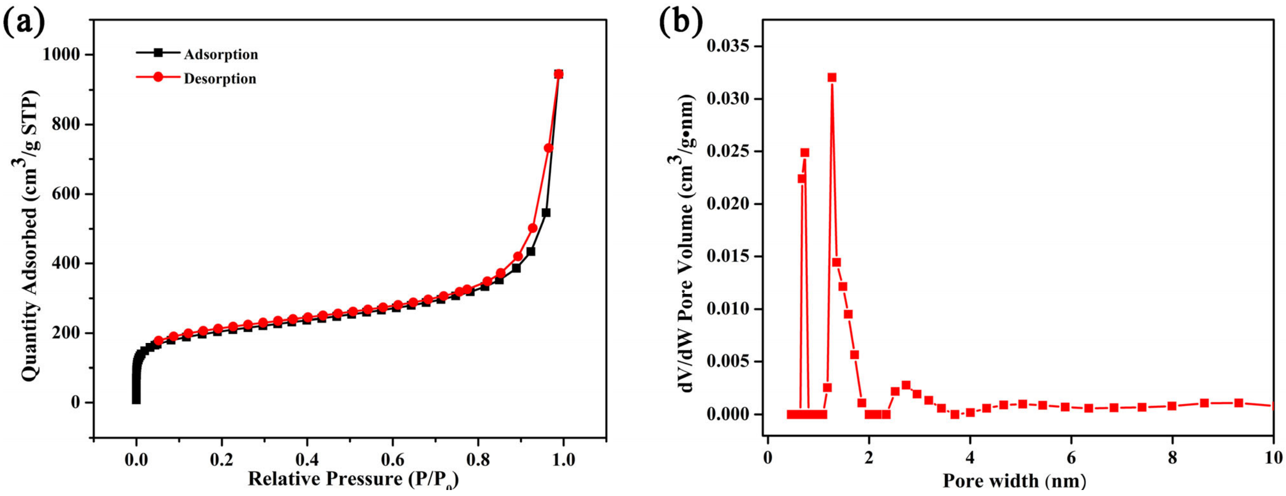

Fig.S6 (a) N_2 adsorption-desorption isotherms, (b) Pore size distribution histogram of SNW-1

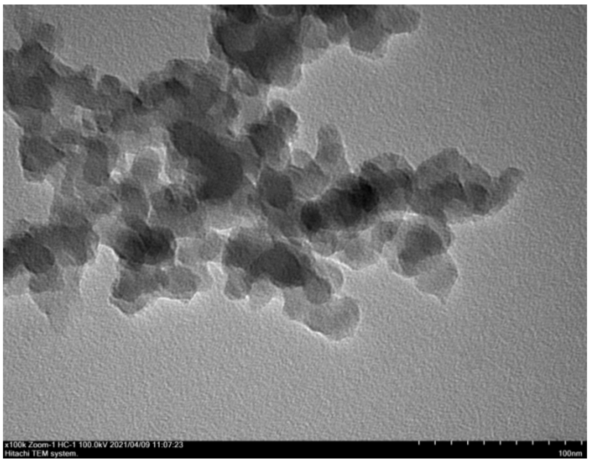

Fig. S7 TEM images SNW-1 nanoparticles

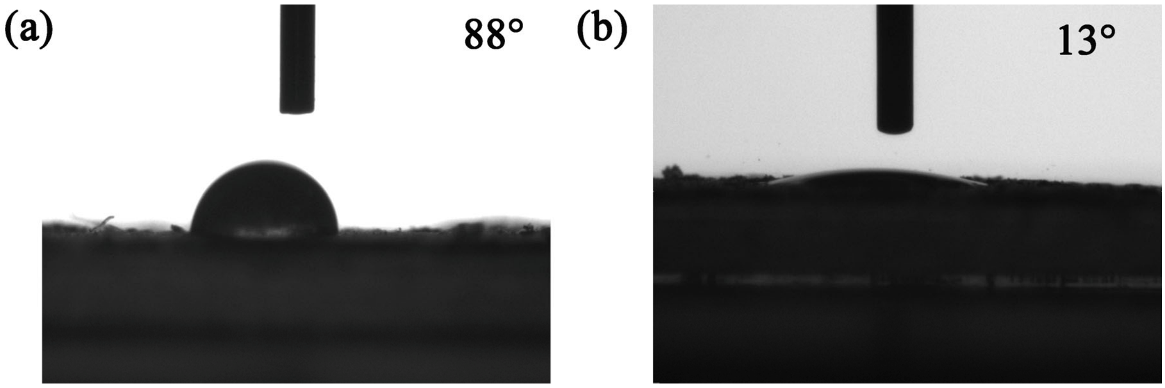

Fig.S8 Water contact angle, (b) Ethanol contact angle of SNW-1 nanoparticles

SNW-1 was characterized by XRD, and the characterization results are shown in **Fig.S5**. At 22°, a characteristic peak appeared nearby, preliminarily proving the synthesis of SNW-1.Subsequently, Subsequently, FTIR was used to explore the chemical structure of SNW-1. In the infrared spectrum of SNW-1, the characteristic peak of p-benzaldehyde (the stretching vibration peak of C=O group at 1700 cm^{-1} and the stretching vibration peak of C-H group at 2700 cm^{-1} and 2800 cm^{-1}) has disappeared, while the peak at 800 cm^{-1} represents the para substitution of benzene ring. The broad peak detected at 3400 cm^{-1} corresponds to the stretching vibration peak of the - NH group, indicating that melamine has been successfully converted, while the peaks at 1548 cm^{-1}, 1465 cm^{-1} and 1357 cm^{-1} represent that triazine rings appear in SNW-1, demonstrating the synthesis of SNW-1.As shown in **Fig.S6**, SNW-1 has a specific surface area of 674 m^2/g and a pore size of about 0.7 nm. It can be seen from the TEM of **Fig.S7** that SNW-1 also presents a lamellar structure, and the contact angle test results of **Fig.S8** also show excellent amphiphilicity. It is also due to the presence of a large number of triazine rings and amino groups.

Characterization of SCF-FCOF-2 filler

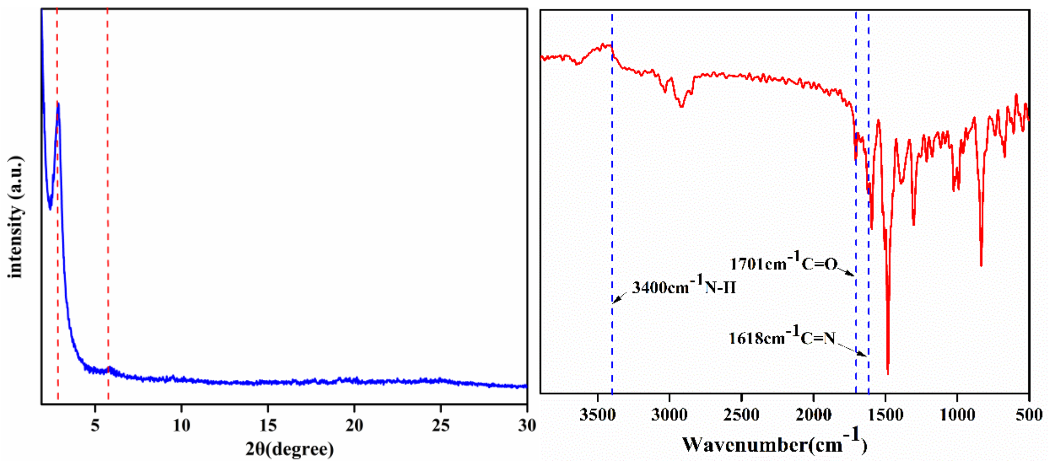

Fig.S9 XRD spectra and FTIR spectra of SCF-FCOF-2 nanoparticles

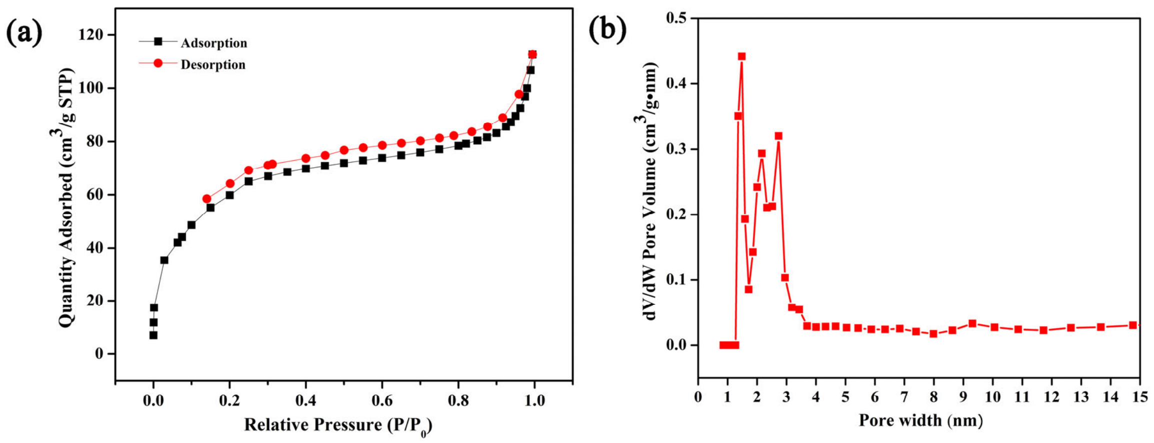

Fig.S10 (a) N_2 adsorption-desorption isotherms, (b) Pore size distribution histogram of SCF-FCOF-2

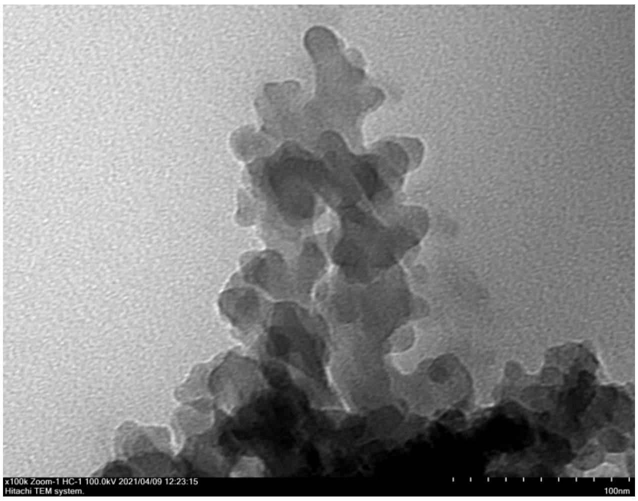

Fig. S11 TEM images of SCF-FCOF-2 nanoparticles

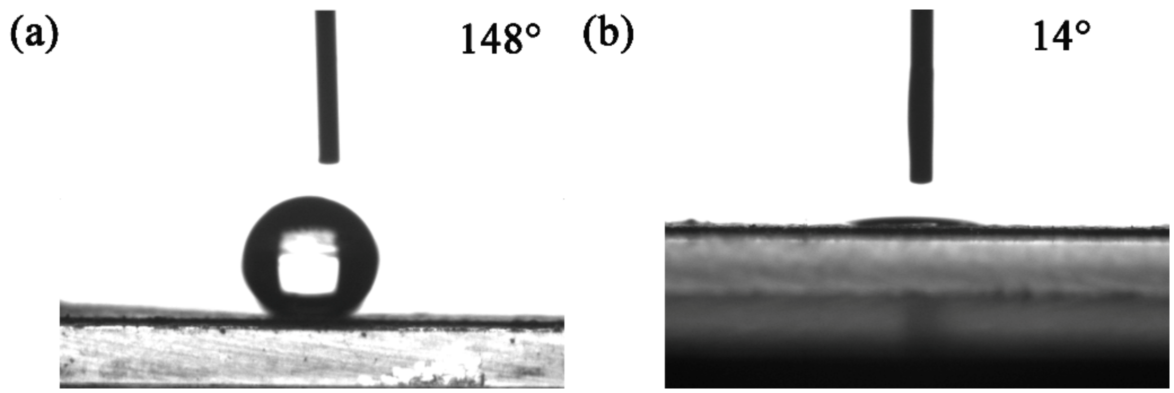

Fig.S12 Water contact angle, (b) Ethanol contact angle of SCF-FCOF-2 nanoparticles

The SCF-FCOF-2 was characterized by XRD, and the characterization results are shown in **Fig.S9**. At 2.93°, a sharp (100) crystal surface characteristic peak appeared, preliminarily proving the synthesis of the SCF-FCOF-2 filler. Then, the chemical structure of SCF-FCOF-2 was characterized by FTIR. In the infrared spectrum of SCF-FCOF-2, the stretching vibration peak of $-NH_2$ group at 3400 cm^{-1} did not appear, indicating that the amino group on 1,3,5-tris (4-aminophenyl) benzene had reacted, while the characteristic peak at 1618 cm^{-1} represented the weakening of the stretching vibration peak of C=N and the stretching vibration peak at 1701 cm^{-1} represented the strength of C=O. This fully demonstrated the generation of imino groups on SCF-FCOF-2 and further verified the successful synthesis of SCF-FCOF-2. The SCF-FCOF-2 was characterized by BET, as shown in **Fig.S10**: the specific surface area was found to be 224 m^2/g, and the pore size was about 3.1 nm. It can be clearly seen from the TEM of **Fig.S11** that SCF-FFCOF 2 also presents a sheet-like stack structure, and the contact angle test results of **Fig.S12** also show its excellent hydrophobicity. This is due to the presence of a large number of benzene rings and the fluorine inherited from the tetrafluoro-p-phenyldiformaldehyde benzene ring.

Characterization of SCF-FCOF-2x filler

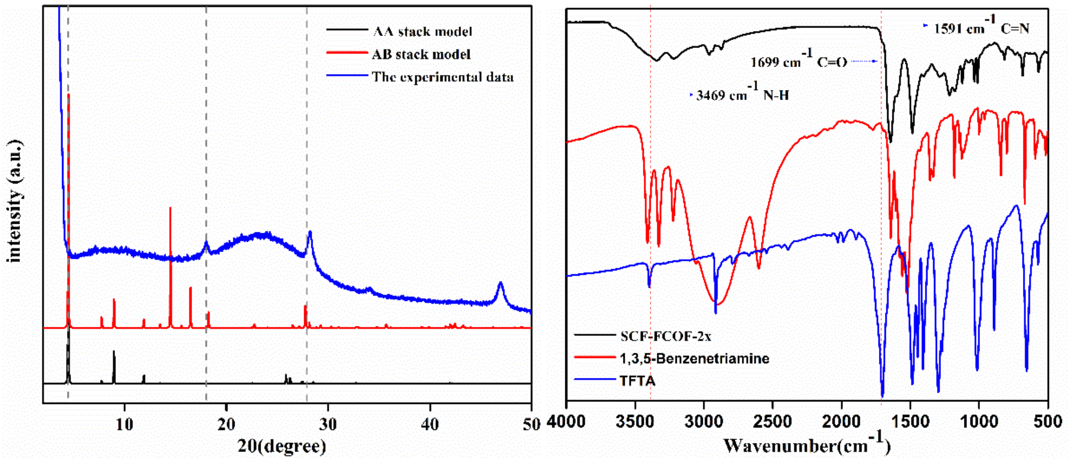

Fig.S13 XRD spectra and FTIR spectra of SCF-FCOF-2x nanoparticles

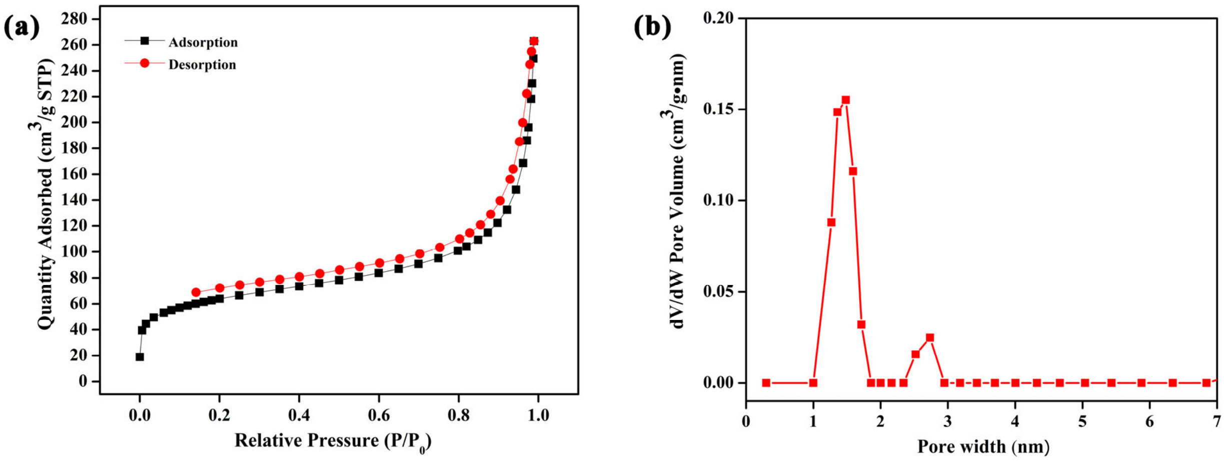

Fig.S14 (a) N_2 adsorption-desorption isotherms, (b) Pore size distribution histogram of SCF-FCOF-2x

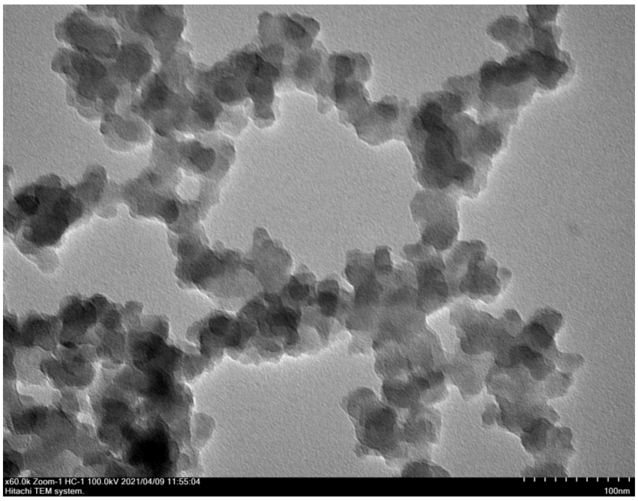

Fig.S15 SEM images of SCF-FCOF-2x nanoparticles

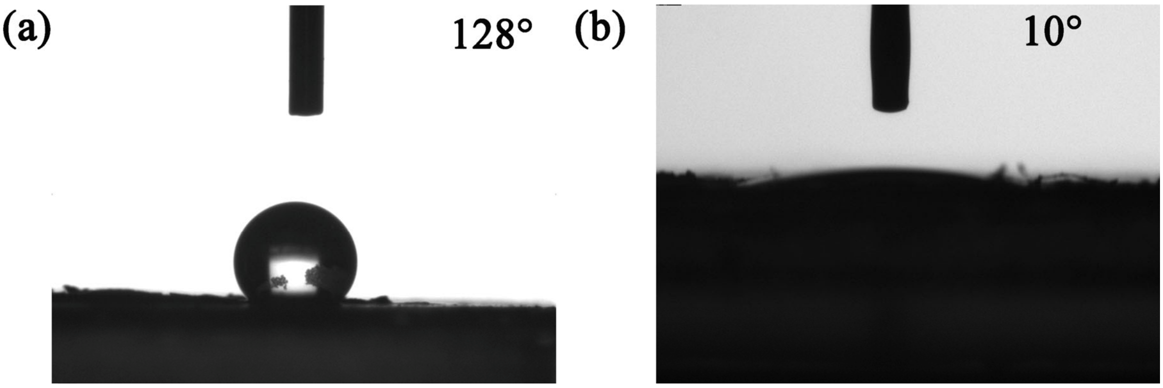

Fig. S16 (a) Water contact angle, (b) Ethanol contact angle of SCF-FCOF-2x nanoparticles

The XRD characterization of SCF-FCOF-2x, as shown in **Fig.S13**, reveals that compared with the simulated structure, the characteristic peaks of the synthesized SCF-FCOF-2x appear at 18.8° and 28.1°, similar to the XRD of the AB stacked simulation model structure, indicating certain crystallinity. However, the characteristic peaks at 4.45° do not appear in the experimentally synthesized SCF-FCOF-2x, possibly due to the staggered single crystal layers that prevent the formation of AA or AB stacks, resulting in poor crystallinity. Then, the chemical structure of SCF-FCOF-2x was characterized by FTIR. The stretching vibration peak representing -NH- at 3469 cm^{-1} has been significantly weakened, and the peak representing C=O at 1699 cm^{-1} has almost disappeared, indicating the reaction between tetrafluoro-p-benzaldehyde and 1,3,5-triaminobenzene trihydrochloride. The new characteristic peak at 1591 cm^{-1} represents the stretching vibration peak of C=N, signifying the generation of imino groups and further demonstrating the successful synthesis of SCF-FCOF-2x.SCF-FCOF-2x was also characterized by BET, as shown in **Fig.S14**, with a specific surface area of 218 m^2/g and a pore size of about 1.4 nm. It can be clearly seen from the TEM of **Fig.S15** that SCF-FCOF-X2 also presents a sheet-like stack structure, and the contact angle test results of **Fig.S16** show that SCF-FCOF-X2 has a strong affinity and hydrophobicity for ethanol.

S3. Characterization of COFs/PDMS MMMs

Characterization of NENP-1 / PDMS mixed matrix membrane

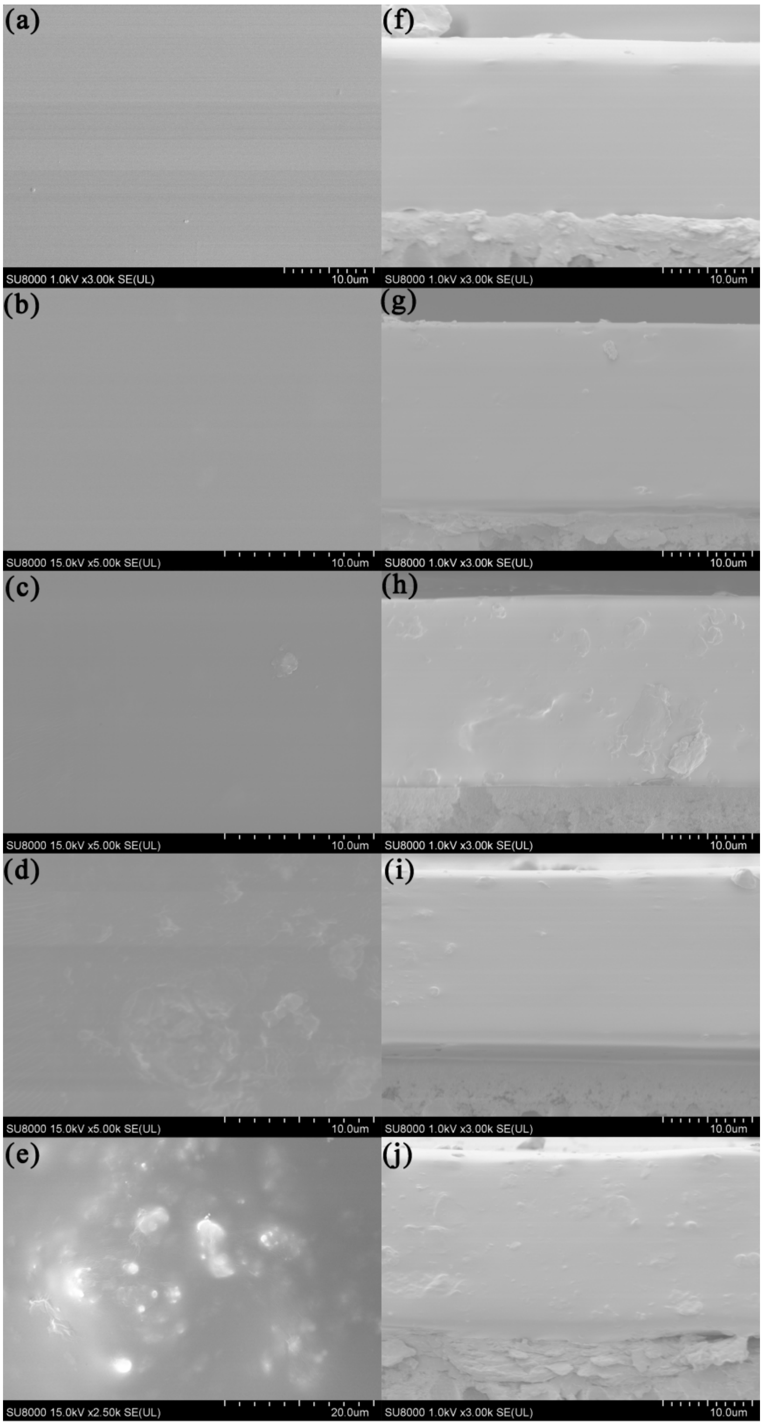

Fig. S17. Surface and cross section of pure PDMS membrane and NENP-1/PDMS mixed matrix membrane with different NENP-1nanoparticle loading a, f, 0 wt.%; b, g, 1 wt.%; c, h, 3 wt.%; d, i, 5 wt.%; e, j, 7 wt.%.

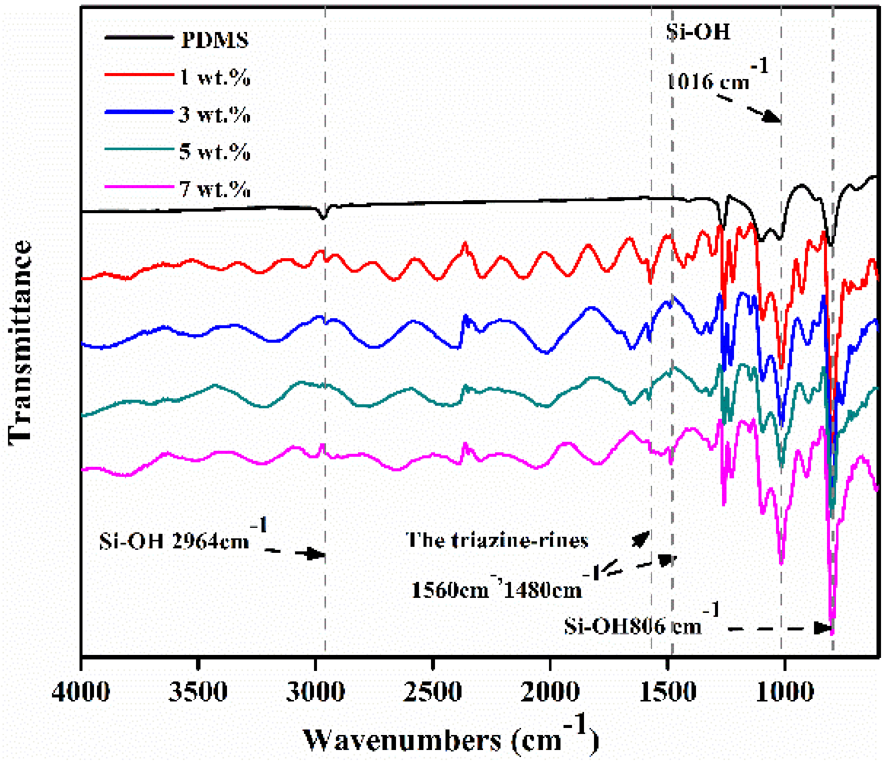

Fig.S18. FTIR spectra of pristine PDMS and NENP-1/PDMS mixed matrix membrane with different NENP-1 nanoparticle loadings.

FTIR was used to characterize the chemical structures of PDMS pure films and NENP-1/PDMS mixed matrix films with different doping amounts. As shown in **Fig.S18**, the absorption peaks at 2964 cm^{-1}, 1016 cm^{-1} and 806 cm^{-1} belong to Si-OH stretching vibration peaks. After adding NENP-1, with the increase of doping amount, the absorption peaks at 1560 cm^{-1} and 1480 cm^{-1} belong to the stretching vibration peaks of triazine ring. It is consistent with the stretching vibration peak of triazine ring in the infrared spectrum of NENP-1.

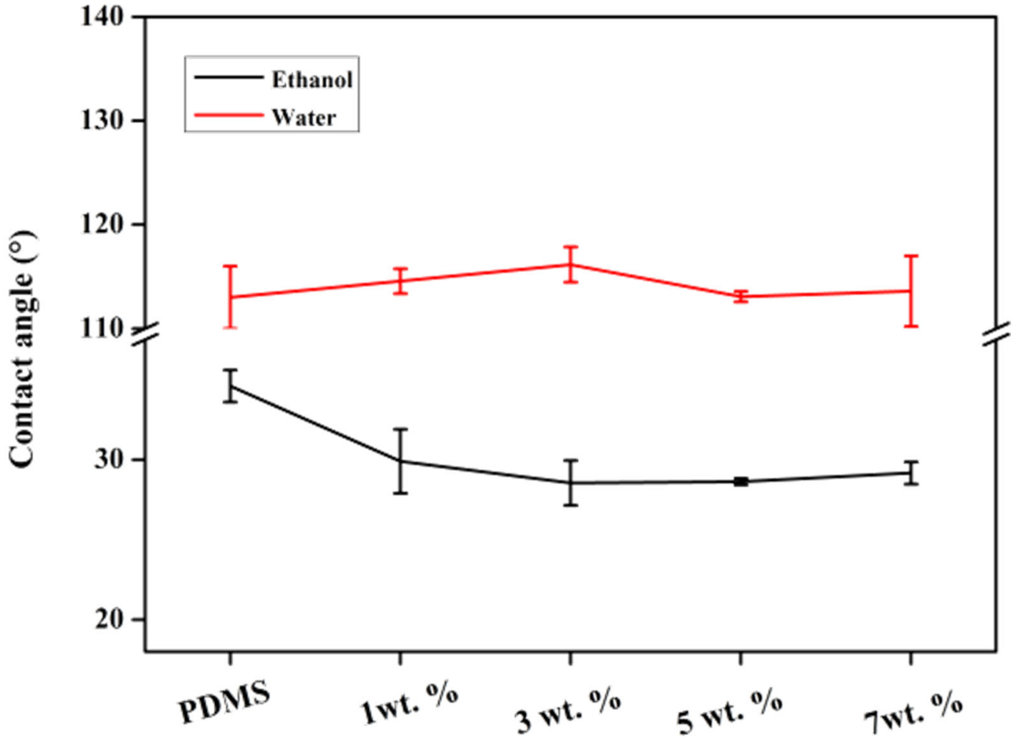

Fig. S19 Water contact angles of pristine PDMS membrane and NENP-1/PDMS mixed matrix membrane.

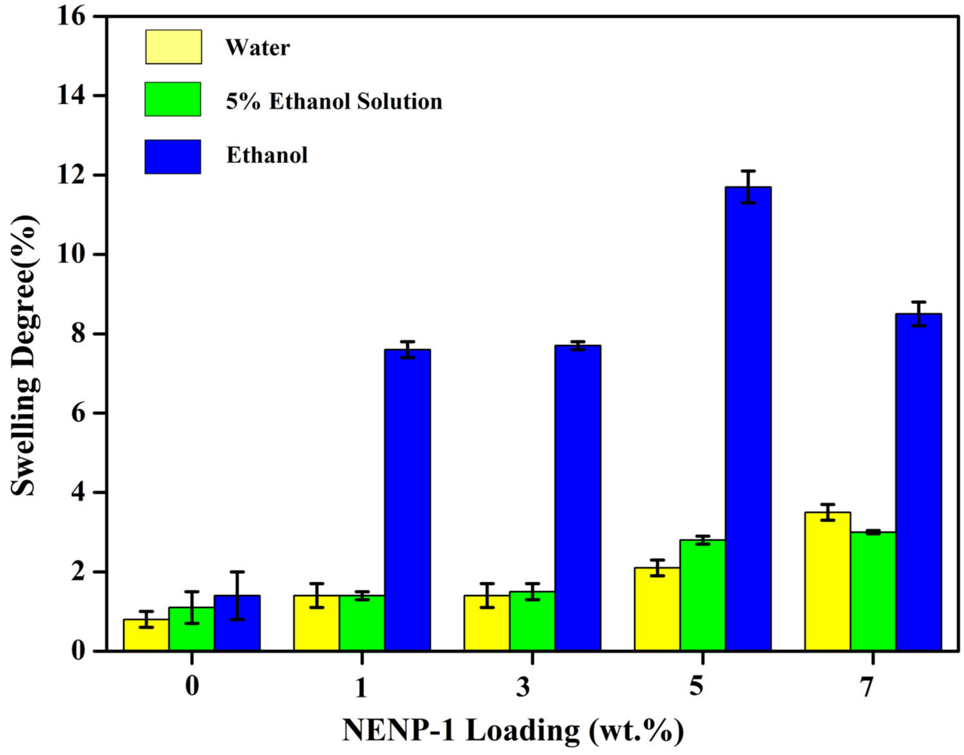

Fig.S20 Swelling degrees of pristine PDMS and NENP-1/PDMS mixed matrix membrane.

The hydrophilicity and hydrophobicity of the mixed matrix membrane surface are closely related to the dissolution of ethanol and water molecules. The change of

hydrophilicity and hydrophobicity of the membrane surface is characterized by the contact angle. As shown in **Fig.S19**, with the increase of doping amount, the hydrophobicity of the membrane surface changes little, only slightly increases at 3 wt.%, while the contact angle to ethanol decreases slowly. It shows that the affinity for ethanol has been improved to a certain extent. As shown in **Fig.S20**, when the doping amount is less than 5 wt.%, the swelling degree in water, ethanol and 5 wt.% ethanol solution increases to varying degrees with the increase of doping amount, among which the increase of ethanol is the most obvious. When the doping amount is 5 wt.%, the swelling degree of ethanol in NENP-1/PDMS mixed matrix membrane increases by 735.7% compared with that of pure membrane. When the doping amount is greater than 5 wt.%, it may be due to a large number of agglomeration, resulting in interface defects and affecting the swelling degree of ethanol.

Characterization of SNW-1 / PDMS mixed matrix membrane

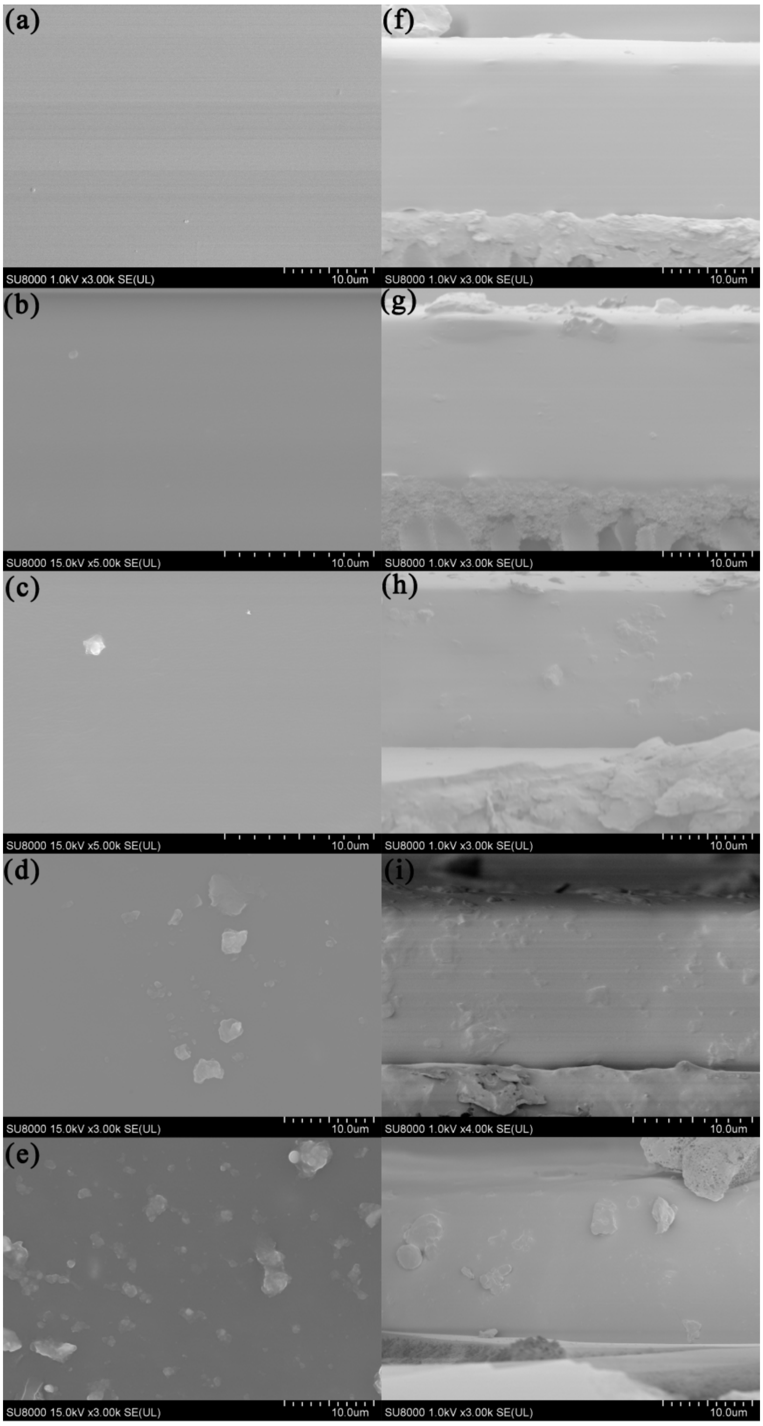

Fig.S21. Surface and cross section of pure PDMS membrane and SNW-1/PDMS mixed matrix membrane with different NENP-1nanoparticle loading a, f, 0 wt.%; b, g, 1 wt.%; c, h, 3 wt.%; d, i, 5 wt.%; e, j, 7 wt.%.

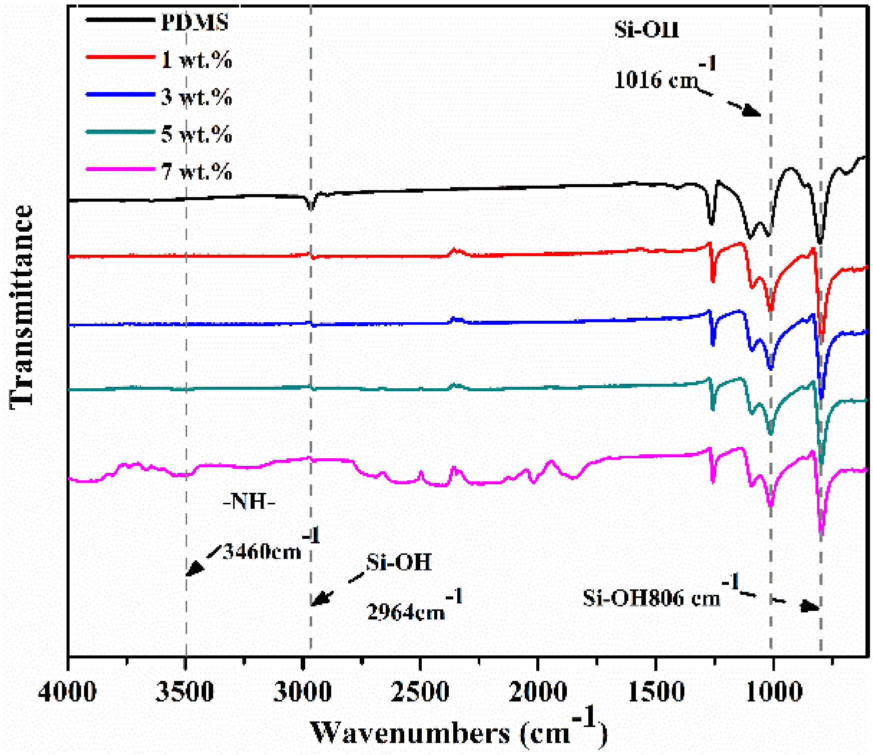

Fig.S22. FTIR spectra of pristine PDMS and NENP-1/PDMS mixed matrix membrane with different SNW-1 nanoparticle loadings.

FTIR was used to characterize the chemical structure of PDMS pure film and SNW-1 / PDMS mixed matrix film with different doping amounts. As shown in **Fig.S22**, with the continuous increase of SNW-1 doping amount, the stretching vibration peak of Si-OH at 1016 cm^{-1} and 806 cm^{-1} was not significantly affected, while the stretching vibration peak of Si-OH at 2964 cm^{-1} was disturbed and disappeared. A new absorption peak appeared at 3460 cm^{-1}, representing the stretching vibration peak of -NH- group in SNW-1. Similarly, the change of hydrophilicity and hydrophobicity on the membrane surface was characterized by the contact angle.

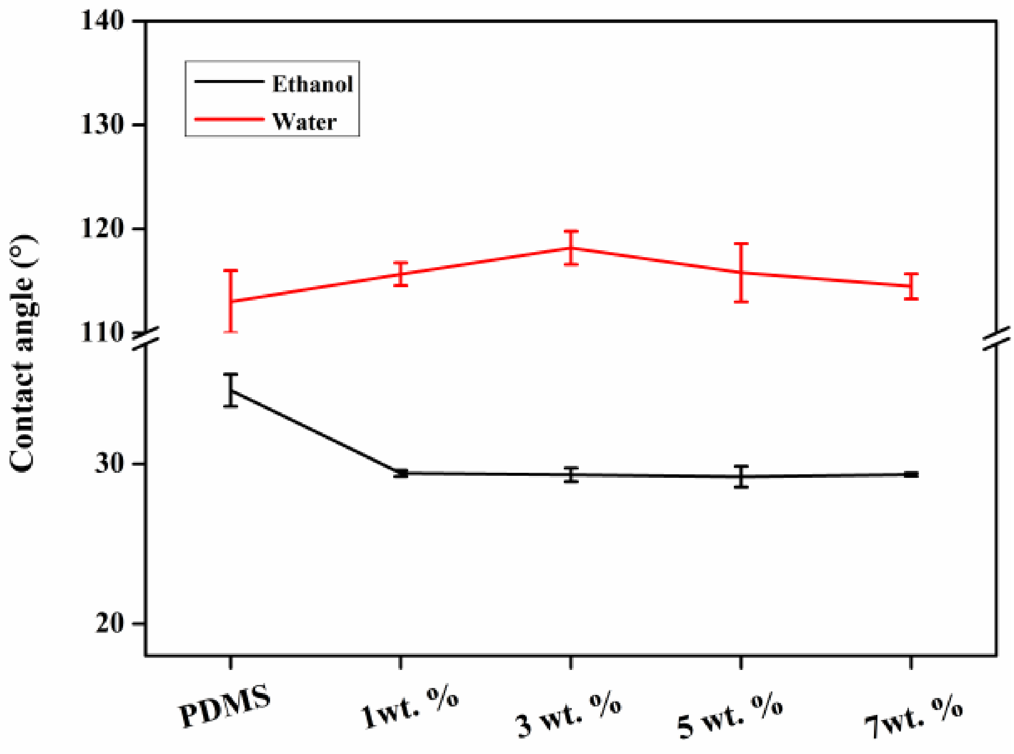

Fig.S23. Water contact angles of pristine PDMS membrane and SNW-1/PDMS mixed matrix membrane.

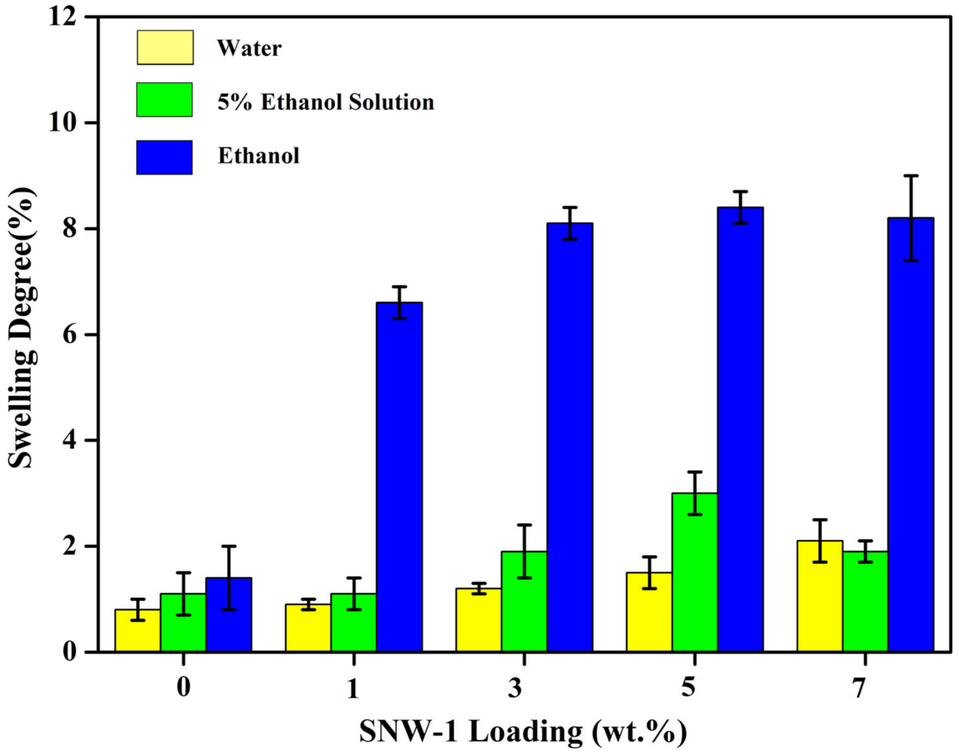

Fig.S24. Swelling degrees of pristine PDMS and SNW-1/PDMS mixed matrix membrane.

As shown in **Fig. S23**, the hydrophobicity of the membrane surface increased when the doping amount reached 3 wt.%, while the contact angle with ethanol decreased gradually,

indicating an improved affinity for ethanol. The swelling degree was also evaluated. As illustrated in **Fig. S24**, when the doping amount was less than 5 wt.%, the swelling degree in water, ethanol, and a 5 wt.% ethanol solution increased to varying extents with the increase in doping amount, with the most significant increase observed in ethanol. Specifically, when the doping amount reached 5 wt.%, the swelling degree of ethanol in the SNW-1/PDMS mixed matrix membrane increased by 500% compared to the pure membrane. However, when the doping amount exceeded 5 wt.%, the excessive aggregation likely caused interface defects, which affected the swelling behavior in ethanol.

Characterization of SCF-FCOF-2 mixed matrix membrane

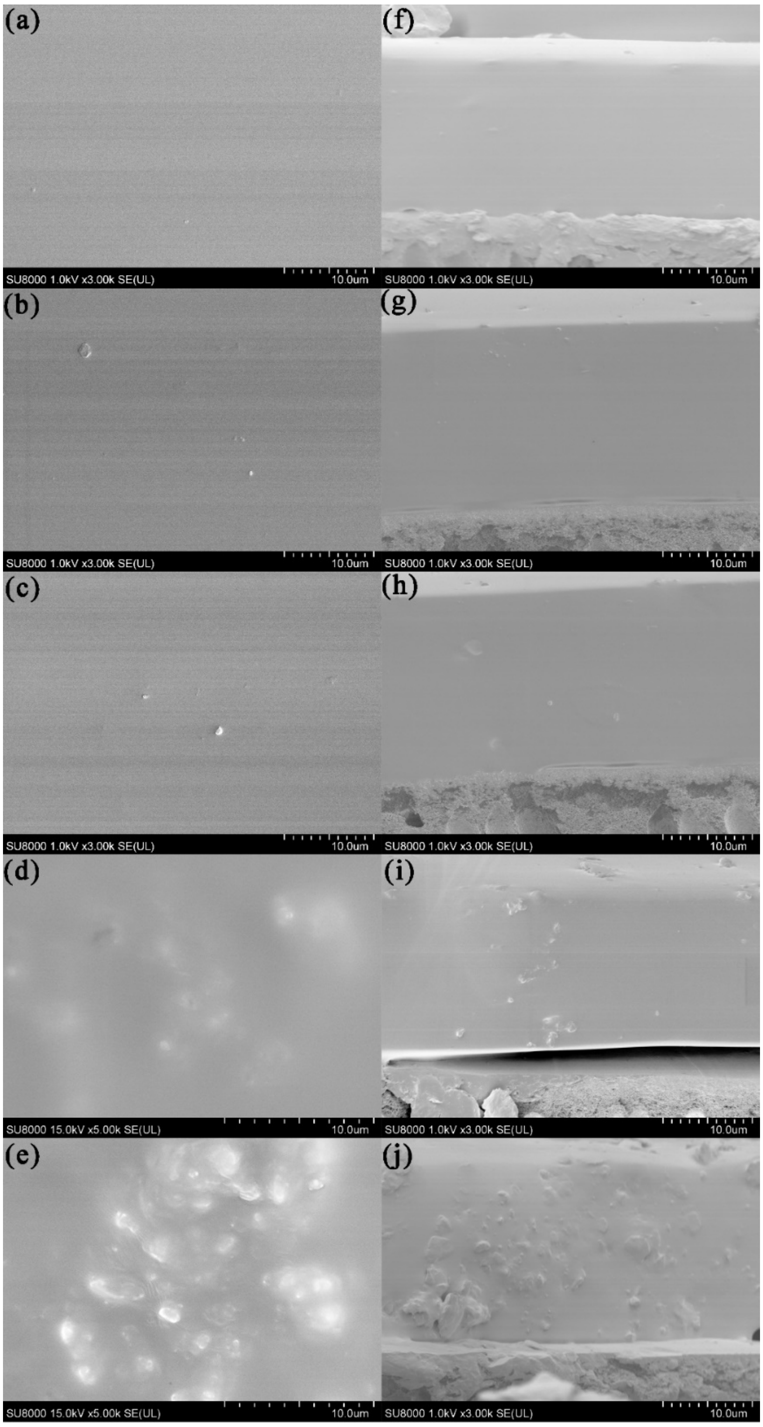

Fig.S25. Surface and cross section of pure PDMS membrane and SCF-FCOF-2/PDMS mixed matrix membrane with different SCF-FCOF-2 nanoparticle loading a, f, 0 wt.%; b, g, 1 wt.%; c, h, 3 wt.%; d, i, 5 wt.%; e, j, 7 wt.%.

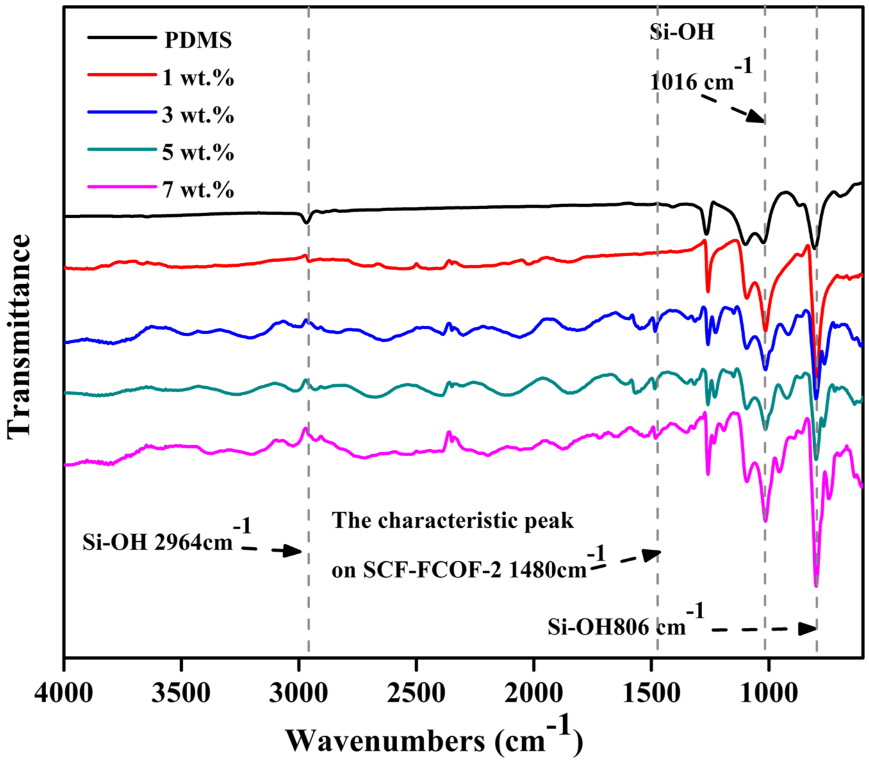

Fig. S26. FTIR spectra of pristine PDMS and SCF-FCOF-2/PDMS mixed matrix membrane with different NENP-1 nanoparticle loadings.

FTIR was employed to characterize the chemical structures of pure PDMS films and SCF-FCOF-2/PDMS mixed matrix membranes with varying doping amounts. As shown in **Fig. S26**, the absorption peaks at 2964 cm^{-1}, 1016 cm^{-1}, and 806 cm^{-1} correspond to the Si-OH stretching vibration peaks. Upon the addition of SCF-FCOF-2, a new absorption peak emerges at 1480 cm^{-1}, which intensifies with the increasing doping amount. This peak is consistent with the strongest characteristic peak observed at 1480 cm^{-1} in the infrared spectrum of SCF-FCOF-2. Additionally, the C=N bond stretching vibration peak at 1618 cm^{-1}, which is present in the SCF-FCOF-2 spectrum, may be overshadowed by the PDMS stretching vibration peak due to its lower intensity.

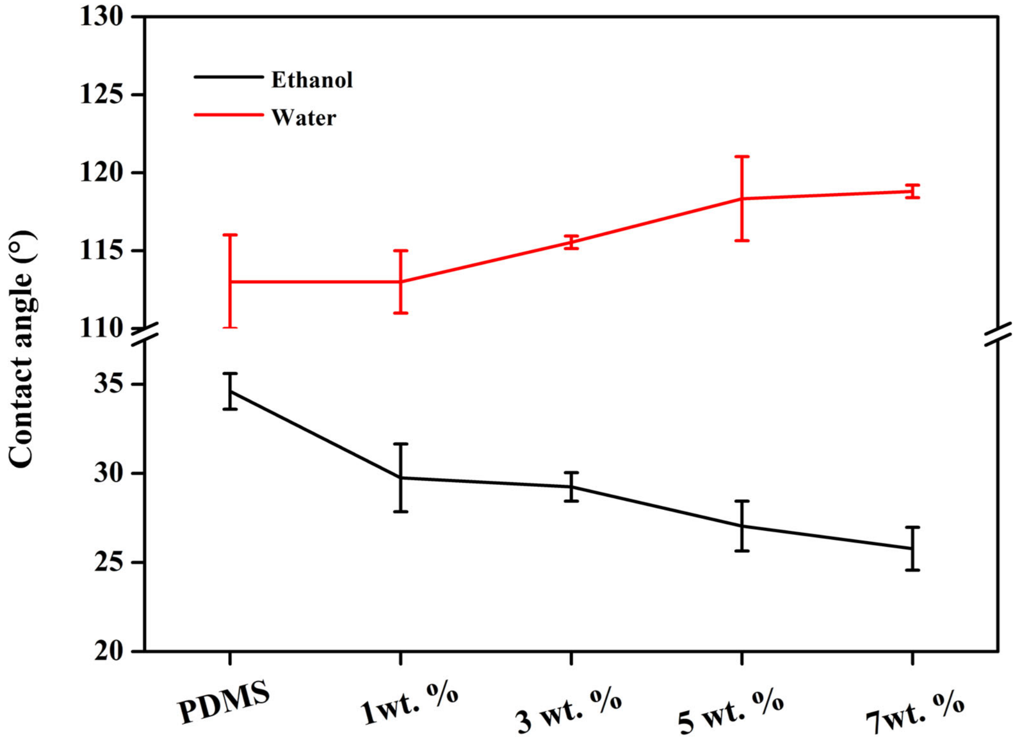

Fig. S27. Water contact angles of pristine PDMS membrane and SCF-FCOF-2/PDMS mixed matrix membrane.

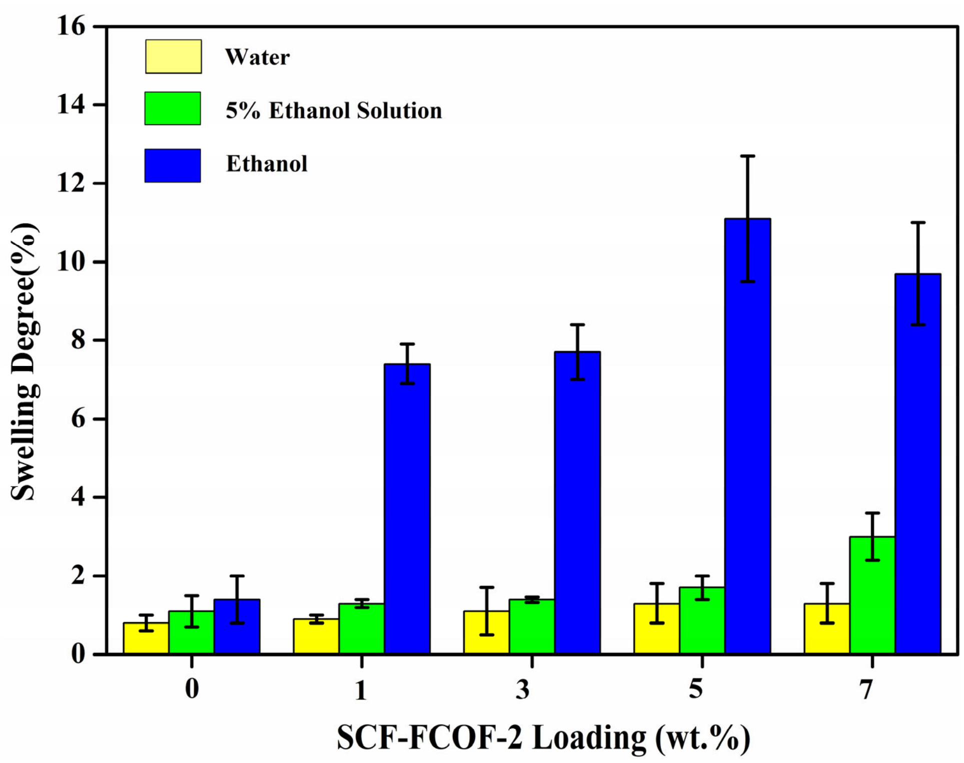

Fig. S28. Swelling degrees of pristine PDMS and SCF-FCOF-2/PDMS mixed matrix membrane.

The hydrophilicity and hydrophobicity of the mixed matrix membrane surface are

closely linked to the dissolution behavior of ethanol and water molecules. These properties are characterized by the contact angle. As shown in **Fig. S27**, the contact angle reached 121.01° with water increases as the doping amount rises, indicating a significant increase in hydrophobicity. Conversely, the contact angle reflected an enhanced affinity for ethanol with increasing ethanol doping. As depicted in **Fig. S28**, when the doping amount is less than 5 wt.%, the swelling degree in water, ethanol, and a 5 wt.% ethanol solution increases to varying extents with higher doping concentrations. The swelling degree in water and 5 wt.% ethanol solution shows only minor changes, while the increase in swelling degree in ethanol is particularly pronounced. At a doping amount of 5 wt.%, the swelling degree of ethanol in the SCF-FCOF-2/PDMS mixed matrix membrane is 692.9% higher than that of the pure membrane. However, when the doping amount exceeds 5 wt.%, the swelling degree tends to decrease, likely due to excessive agglomeration, which creates interface defects that hinder ethanol swelling.

Characterization of SCF-FCOF-2x mixed matrix membrane

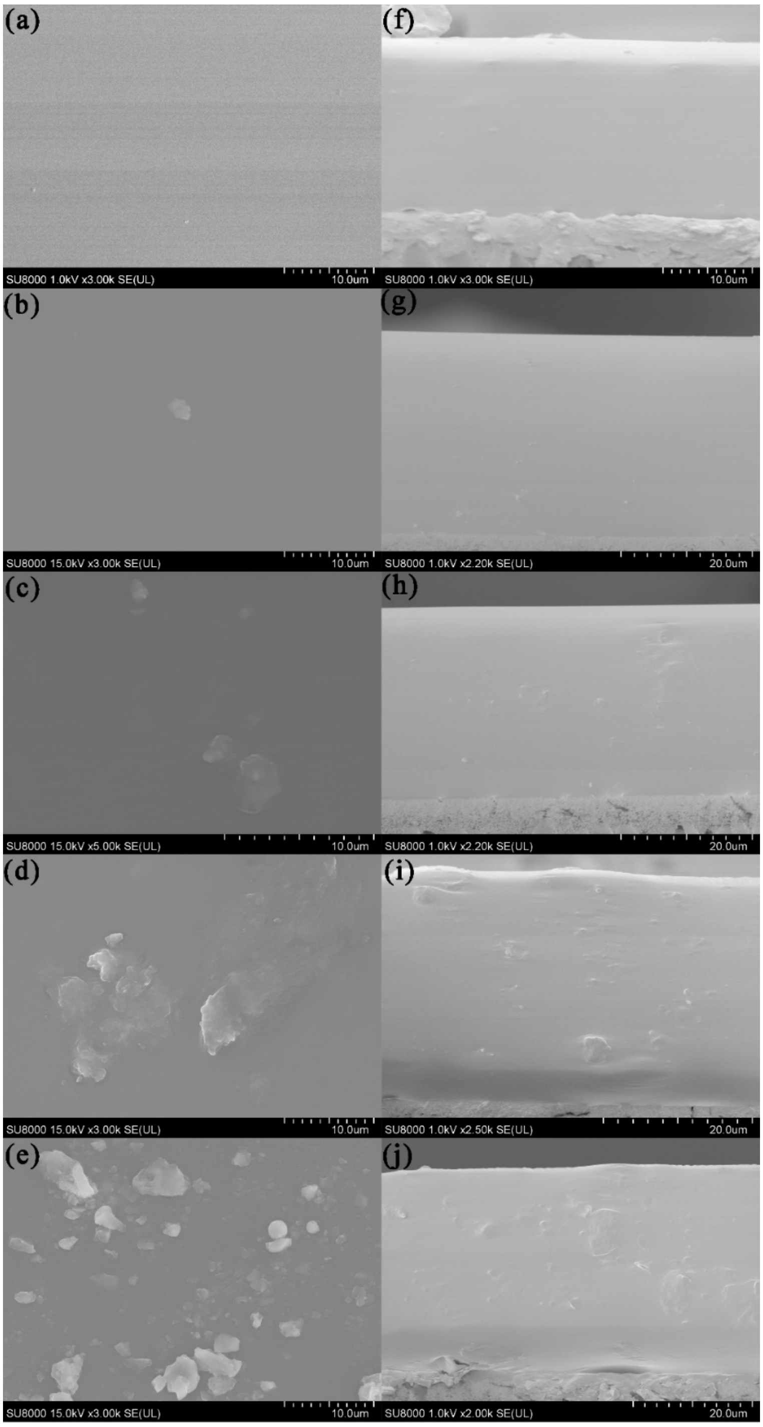

Fig.S29 Surface and cross section of pure PDMS membrane and SCF-FCOF-2x/PDMS mixed matrix membrane with different SCF-FCOF-2x nanoparticle loading a, f, 0 wt.%; b, g, 1 wt.%; c, h, 3 wt.%; d, i, 5 wt.%; e, j, 7 wt.%

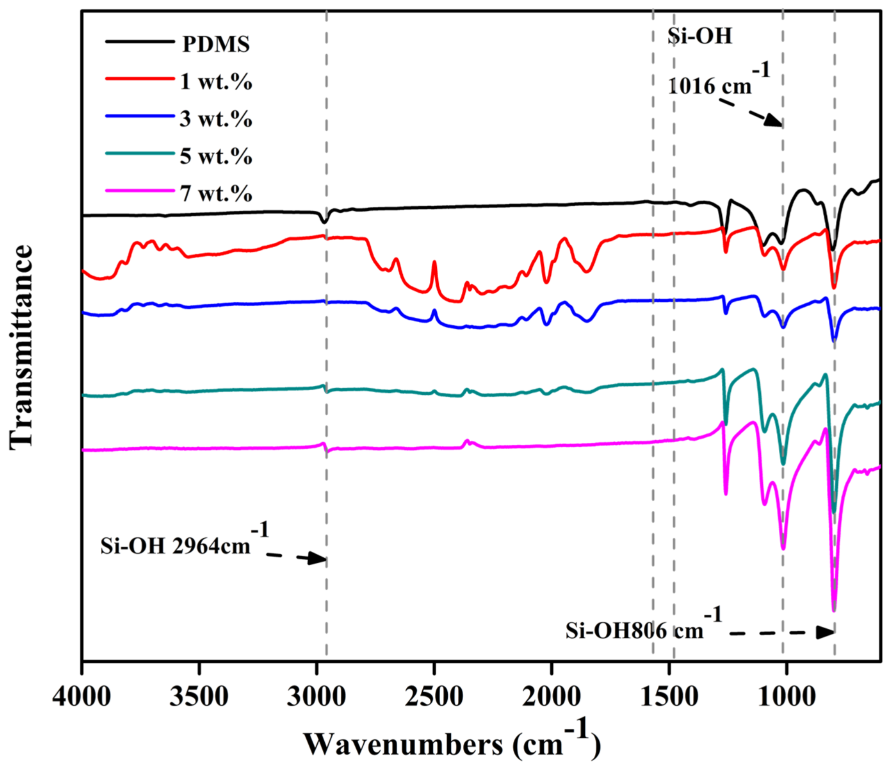

Fig.S30. FTIR spectra of pristine PDMS and SCF-FCOF-2x/PDMS mixed matrix membrane with different SCF-FCOF-2x nanoparticle loadings

FTIR was employed to investigate the chemical structures of pure PDMS films and NENP-1/PDMS mixed matrix films with varying doping amounts. As shown in **Fig. S29**, the absorption peaks at 2964 cm^{-1}, 1016 cm^{-1}, and 806 cm^{-1} correspond to the stretching vibrations of Si-OH. Upon the addition of SCF-FCOF-2x, no significant changes in these characteristic peaks were observed with increasing doping concentrations. This suggests that SCF-FCOF-2x does not notably alter the infrared spectrum, likely because its low absorption peak intensity is overshadowed by the stronger absorption signals from PDMS.

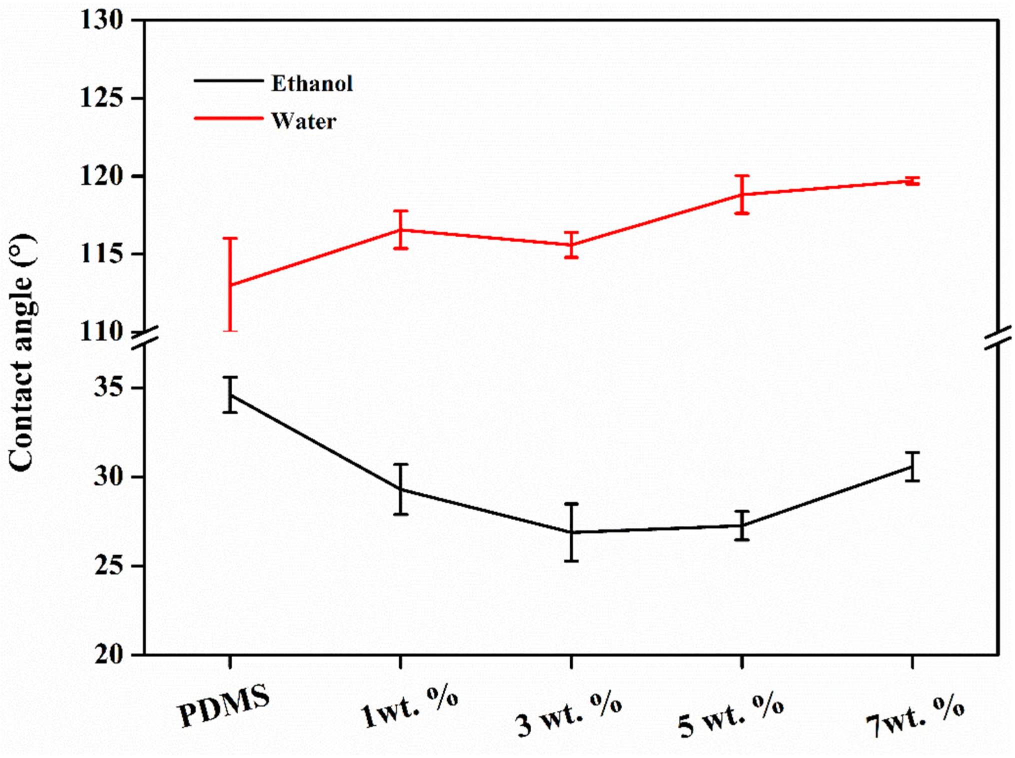

Fig. S30 Water contact angles of pristine PDMS membrane and SCF-FCOF-2x/PDMS mixed matrix membrane

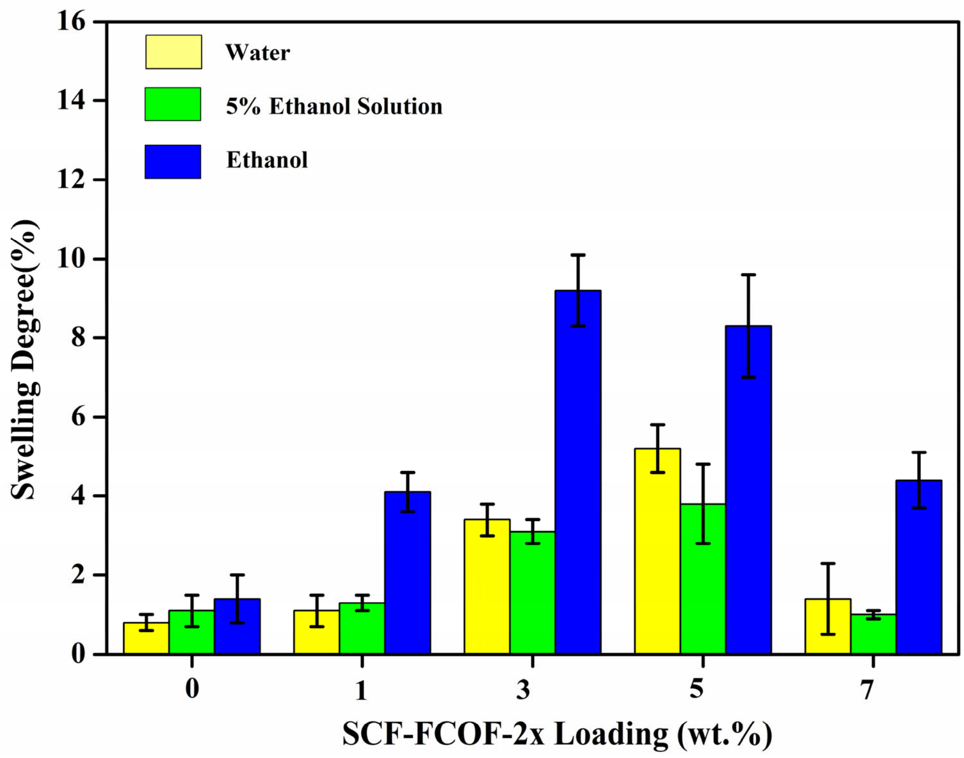

Fig.S31 Swelling degrees of pristine PDMS and SCF-FCOF-2x/PDMS mixed matrix membrane

The hydrophilicity and hydrophobicity of the mixed matrix membrane surface play a

crucial role in the dissolution behavior of ethanol and water molecules. The variation in these properties is characterized by the contact angle measurements. As shown in **Fig.S30**, the water contact angle of the SCF-FCOF-2x/PDMS mixed matrix membrane increases gradually with increasing doping concentration, reaching 120.01° at a doping level of 7 wt.%. This indicates a notable increase in hydrophobicity. Conversely, the contact angle for ethanol decreases initially as the doping amount increases. However, when the doping concentration exceeds 5 wt.%, the contact angle increases slowly, which may be attributed to agglomeration caused by excessive doping, affecting the membrane's surface morphology and, consequently, the interaction of ethanol with the membrane surface.Swelling behavior was also assessed, as shown in **Fig. S31**. When the doping amount was less than 3 wt.%, the swelling degree in water, ethanol, and a 5 wt.% ethanol solution increased with the doping amount, with the most significant swelling observed in ethanol. At 3 wt.%, the swelling degree of ethanol in the SCF-FCOF-2x/PDMS mixed matrix membrane increased by 557.1% compared to that of the pure membrane. However, for doping amounts greater than 3 wt.%, the swelling behavior was less pronounced, likely due to the formation of agglomerates, which introduce interface defects and hinder the swelling of ethanol.

S4. Pervaporation Performance of COFs/PDMS MMMs

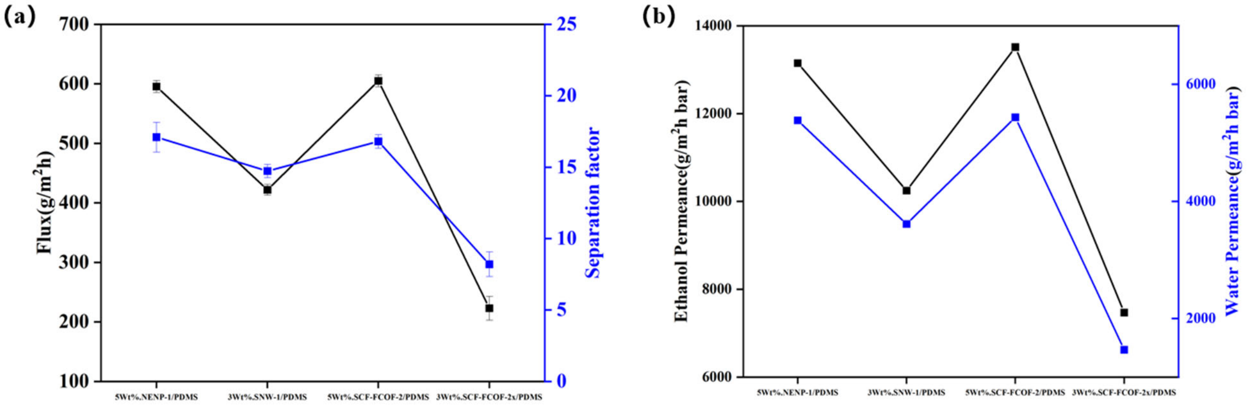

Fig.S32. (a) The PV performances of mixed matrix membrane with different nanoparticle loadings (b)The single component permeances of mixed matrix membrane with different nanoparticle loadings.

Under the condition of 40 °C, the performance of the 5wt% NENP-1/PDMS membrane, 3wt% SNW-1/PDMS membrane, 5wt% SCF-FCOF-2/PDMS membrane, and 3wt% SCF-FCOF-2x/PDMS membrane for separating 5wt% ethanol-water solution is shown in **Fig.S32**(a).At this time, the permeance and selectivity of the 5wt% NENP-1/PDMS mixed matrix membrane are 596 g/(m^2·h) and 17.1, respectively; the permeance and selectivity of the 3wt% SNW-1/PDMS mixed matrix membrane are 422 g/(m^2·h) and 14.7, respectively; the permeance of the 5wt% SCF-FCOF-2/PDMS mixed matrix membrane is 605 g/(m^2·h), and the selectivity reaches 16.8; the permeance of the 3wt% SCF-FCOF-2x/PDMS mixed matrix membrane is 222.93 g/(m^2·h), and the selectivity reaches 8.2. As shown in **Figure S32**(b), the ethanol single-component permeance of the 5wt% NENP-1/PDMS mixed matrix membrane is 18060.9 g/(m^2·h·bar), and the water single-component permeance is 4325.2 g/(m^2·h·bar); the ethanol single-component permeance of the 3wt% SNW-1/PDMS mixed matrix membrane is 11800.9 g/(m^2·h·bar), and the water single-component permeance is

3279.9 g/(m^2·h·bar); the ethanol single-component permeance of the 5wt% SCF-FCOF-2/PDMS mixed matrix membrane is 18178.8 g/(m^2·h·bar), and the water single-component permeance is 4431.2 g/(m^2·h·bar); the ethanol single-component permeance of the 3wt% SCF-FCOF-2x/PDMS mixed matrix membrane is 4303.2 g/(m^2·h·bar), and the water single-component permeance is 2149.1 g/(m^2·h·bar).
